# Supplementary material for: Pervasive misannotation of microexons that are evolutionarily conserved and crucial for gene function in plants
Source: Nat Commun. 2022 Feb 10;13:820. doi: 10.1038/s41467-022-28449-8 (PMC8831610; doi:10.1038/s41467-022-28449-8)
Supplement: Supplementary file 1 — Supplementary Information [file 41467_2022_28449_MOESM1_ESM.pdf]

Pervasive misannotation of microexons that are evolutionarily conserved and crucial for gene function in plants

Huihui Yu, Mu Li, Jaspreet Sandhu, Guangchao Sun, James C. Schnable, Harkamal Walia, Weibo Xie, Bin Yu, Jeffrey P. Mower, Chi Zhang

Supplementary Table 1: Distribution of microexon lengths

	3N+0	3N+1	3N+2	3NT	Non 3NT
C. reinhardtii	164	89	84	0.49	0.51
P. patens	65	30	63	0.41	0.59
S. moellendorffii	136	63	71	0.50	0.50
P. somniferum	190	129	135	0.42	0.58
A. thaliana	65	22	37	0.52	0.48
G. max	157	50	82	0.54	0.46
V. vinifera	93	51	40	0.51	0.49
H. annuus	155	71	76	0.51	0.49
O. sativa	51	26	28	0.49	0.51
Z. mays	73	61	41	0.42	0.58

Supplementary Table 2: Protein motifs encoded by microexons in 10 plant species

Motif	Cases	Percentage (%)	Accession	Description
AP2	127	7.59	PF00847	AP2 domain
Glyco_hydro_32N	92	5.5	PF00251	Glycosyl hydrolases family 32 N-terminal domain
Myosin_head	40	2.39	PF00063	Myosin head (motor domain)
bHLH-MYC_N	28	1.67	PF14215	bHLH-MYC and R2R3-MYB transcription factors N-terminal
Peptidase_M1	23	1.37	PF01433	Peptidase family M1 domain
Gelsolin	22	1.31	PF00626	Gelsolin repeat
PK_Tyr_Ser-Thr	19	1.14	PF07714	Protein tyrosine and serine/threonine kinase
Pkinase	19	1.14	PF00069	Protein kinase domain
Tudor-knot	19	1.14	PF11717	RNA binding activity-knot of a chromodomain
RPE65	18	1.08	PF03055	Retinal pigment epithelial membrane protein
CDP-OH_P_transf	17	1.02	PF01066	CDP-alcohol phosphatidyltransferase
DUF1325	17	1.02	PF07039	SGF29 tudor-like domain
GBP	16	0.96	PF02263	Guanylate-binding protein, N-terminal domain
Myb_DNA-binding	16	0.96	PF00249	Myb-like DNA-binding domain
TPT	16	0.96	PF03151	Triose-phosphate Transporter family
Ribul_P_3_epim	15	0.9	PF00834	Ribulose-phosphate 3 epimerase family
TRAP_beta	15	0.9	PF05753	Translocon-associated protein beta (TRAPB)
PRMT5	14	0.84	PF05185	PRMT5 arginine-N-methyltransferase
Trigger_N	14	0.84	PF05697	Bacterial trigger factor protein (TF)
CHORD	12	0.72	PF04968	CHORD
DEAD	12	0.72	PF00270	DEAD/DEAH box helicase
DUF974	12	0.72	PF06159	Protein of unknown function (DUF974)
EFP_N	12	0.72	PF08207	Elongation factor P (EF-P) KOW-like domain
Prefoldin_2	12	0.72	PF01920	Prefoldin subunit
Ham1p_like	11	0.66	PF01725	Ham1 family
Peptidase_C1	11	0.66	PF00112	Papain family cysteine protease
Ribonuclease_T2	11	0.66	PF00445	Ribonuclease T2 family
TPR_12	10	0.6	PF13424	Tetratricopeptide repeat
Helicase_C	9	0.54	PF00271	Helicase conserved C-terminal domain
Guanylate_cyc	8	0.48	PF00211	Adenylate and Guanylate cyclase catalytic domain
IP_trans	8	0.48	PF02121	Phosphatidylinositol transfer protein
RRM_1	8	0.48	PF00076	RNA recognition motif. (a.k.a. RRM, RBD, or RNP domain)
SNF2_N	8	0.48	PF00176	SNF2 family N-terminal domain
Torsin	8	0.48	PF06309	Torsin
Vps55	8	0.48	PF04133	Vacuolar protein sorting 55
DNA_ligase_A_M	7	0.42	PF01068	ATP dependent DNA ligase domain
BUD22	6	0.36	PF09073	BUD22
SPARK	6	0.36	PF19160	SPARK
RCC1	5	0.3	PF00415	Regulator of chromosome condensation (RCC1) repeat
zf-ANAPC11	5	0.3	PF12861	Anaphase-promoting complex subunit 11 RING-H2 finger
GRP	4	0.24	PF07172	Glycine rich protein family
Ion_trans	4	0.24	PF00520	Ion transport protein
PHO4	4	0.24	PF01384	Phosphate transporter family
RCC1_2	4	0.24	PF13540	Regulator of chromosome condensation (RCC1) repeat
AAA_11	3	0.18	PF13086	AAA domain
Ammonium_transp	3	0.18	PF00909	Ammonium Transporter Family
DLH	3	0.18	PF01738	Dienelactone hydrolase family
Metallophos	3	0.18	PF00149	Calcineurin-like phosphoesterase
Nucleotid_trans	3	0.18	PF03407	Nucleotide-diphospho-sugar transferase
Ole_e_6	3	0.18	PF09253	Pollen allergen Ole e 6
Thioredoxin	3	0.18	PF00085	Thioredoxin
VSP	3	0.18	PF03302	Giardia variant-specific surface protein
Other	297	17.74		
Unknown	601	35.9		
Total	1674	100		

Supplementary Table 3: Misannotated coding microexons

	**Unannotated coding microexons in the discovered**			**Unsupported coding microexons in annotations**		
	Number of unannotated	Number of discovered	Unannotated (%)	Number of unsupported	Number of annotated	Unsupported (%)
Arabidopsis	29	106	27.4	46	123	37.4
Soybean	54	235	23.0	269	450	59.8
Rice	68	84	81.0	167	183	91.3
Maize	82	126	65.1	534	578	92.4

Supplementary Table 4: Comparing unannotated discovered-coding-microexons with annotations and their effect on protein length

	Number of unannotated	Nonoverlap with annotated exons	Annotated as Noncoding region	Annotation protein Shorter	Annotation protein Equal	Annotation protein Longer
Arabidopsis	29	2	2	0	0	0
Soybean	54	7	26	11	1	7
Rice	68	1	53	37	9	6
Maize	82	8	53	30	3	12

Supplementary Table 5: Information of 45 microexon clusters from RNA-seq data

Cluster	Size	Phase	Motif	Species	Cases	Exons[a]	Blocks[b]	Order[c]	Existing
1	1	1	zf-ANAPC11	3	5	3	19,1,52	2	Land plants
2	1	1	Vps55	5	8	3	19,1,52	2	Vascular plants
3	3	0	CHORD	8	10	3	54,3,51	2	Land plants
4	4	0	Ribonuclease_T2	6	11	3	51,4,53	2	Flowering plants
5	4	2	Helicase_C	8	9	3	53,4,51	2	Vascular plants
6	5	0	Ribul_P_3_epim	9	15	3	51,5,52	2	Land plants
7	5	1	Peptidase_M1	7	12	5	33,19,5,12,39	3	Land plants
8	5	1	Peptidase_C1	7	11	3	52,5,51	2	Land plants
9	5	2	SKG6	4	4	4	19,34,5,50	3	Land plants
10	6	0	DUF4788	9	28	3	51,6,51	2	Green plants
11	6	0	TRAP_beta	8	15	3	51,6,51	2	Vascular plants
12	6	1	Unknown	3	5	3	52,6,50	2	Flowering plants
13	7	0	DNA_ligase_A_M	6	7	4	13,38,7,50	3	Land plants
14	7	1	Prefoldin_2	9	12	3	52,7,49	2	Land plants
15	7	2	GBP	9	16	3	50,7,51	2	Land plants
16	8	1	SNF2_N	6	8	3	49,8,51	2	Land plants
17	8	1	IP_trans	5	7	3	49,8,51	2	Land plants
18	8	1	DEAD	8	11	3	49,8,51	2	Vascular plants
19	8	2	Unknown	6	11	3	50,8,50	2	Land plants
20	8	2	VSP	3	6	4	16,34,8,50	3	Land plants
21	9	1	AP2	9	127	3	49,9,50	2	Land plants
22	9	1	Glyco_hydro_32N	9	90	3	49,9,50	2	Land plants
23	10	0	AAA	9	10	3	48,10,50	2	Land plants
24	10	1	Ham1p_like	8	11	4	49,10,32,17	2	Land plants
25	10	2	CDP-OH_P_transf	9	17	3	50,10,48	2	Land plants
26	11	1	PRMT5	9	15	3	49,11,48	2	Land plants
27	11	1	Gelsolin	8	47	3	49,11,48	2	Land plants
28	12	0	Peptidase_M1	6	9	5	24,19,5,12,48	4	Land plants
29	12	0	TPR_12	6	9	3	48,12,48	2	Land plants
30	12	1	SPARK	7	11	3	49,12,47	2	Land plants
31	13	0	TPT	8	14	3	48,13,47	2	Land plants
32	13	0	MCM6_C	8	19	3	48,13,47	2	Land plants
33	13	2	Unknown	9	14	3	47,13,48	2	Land plants
34	14	0	DUF1325	9	17	3	48,14,46	2	Land plants
35	14	0	Tudor-knot	7	19	3	48,14,46	2	Vascular plants
36	14	1	EFP_N	8	10	4	15,31,14,48	3	Land plants
37	14	1	Trigger_N	7	14	3	46,14,48	2	Land plants
38	14	1	Myosin_head	9	38	3	46,14,48	2	Land plants
39	14	1	Unknown	5	9	4	24,22,14,48	3	Flowering plants
40	14	1	SBP_bac_10	6	8	3	46,14,48	2	Land plants
41	15	0	DUF974	9	12	3	48,15,45	2	Land plants
42	15	0	bHLH-MYC_N	8	26	3	48,15,45	2	Land plants
43	15	0	Unknown	3	5	3	48,15,45	2	Flowering plants
44	15	1	Unknown	8	11	3	46,15,47	2	Land plants
45	15	2	RPE65	9	18	3	47,15,46	2	Land plants

[a]The number of exons in the 108-nt microexon-tag; [b]size of exons in the 108-nt microexon-tag; [c]order of microexon in exons of the 108-nt microexon-tag

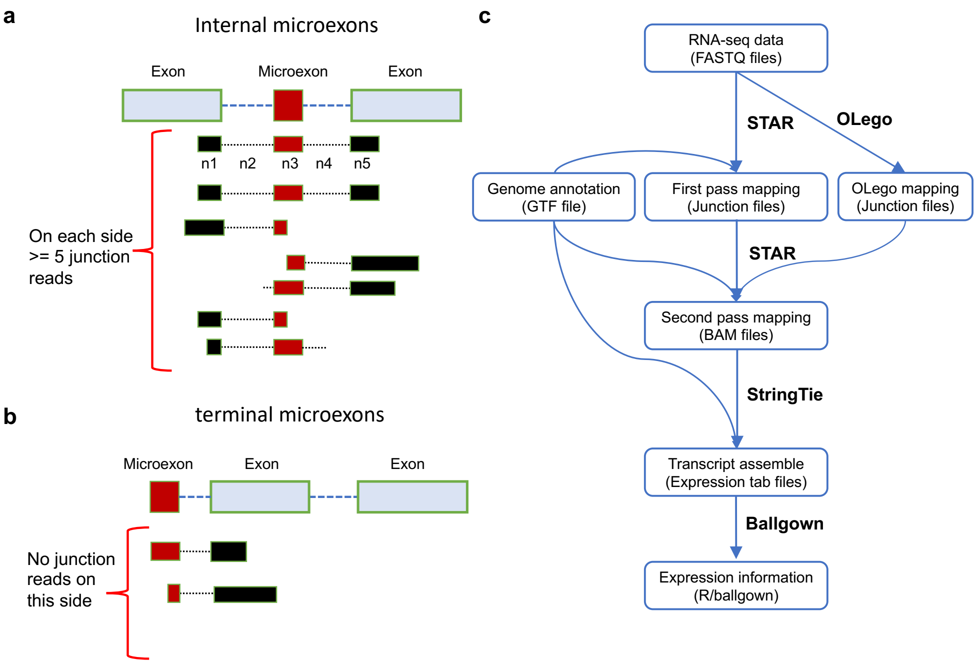

Supplementary Fig. 1: The pipeline of internal microexon discovery in plants. a, Definition of internal microexons and corresponding RNA-seq read alignments (n1-n5 indicates five different parts of a read alignment: n1 is the part of read aligned to the 5' flanking exon, n2 and n4 are two gaps, n3 is the part of read mapped to the internal microexons, and n5 is the part of read aligned to the 3' flanking exon. **b**, Definition of terminal microexons. **c**, Pipeline of microexon identification in plants.

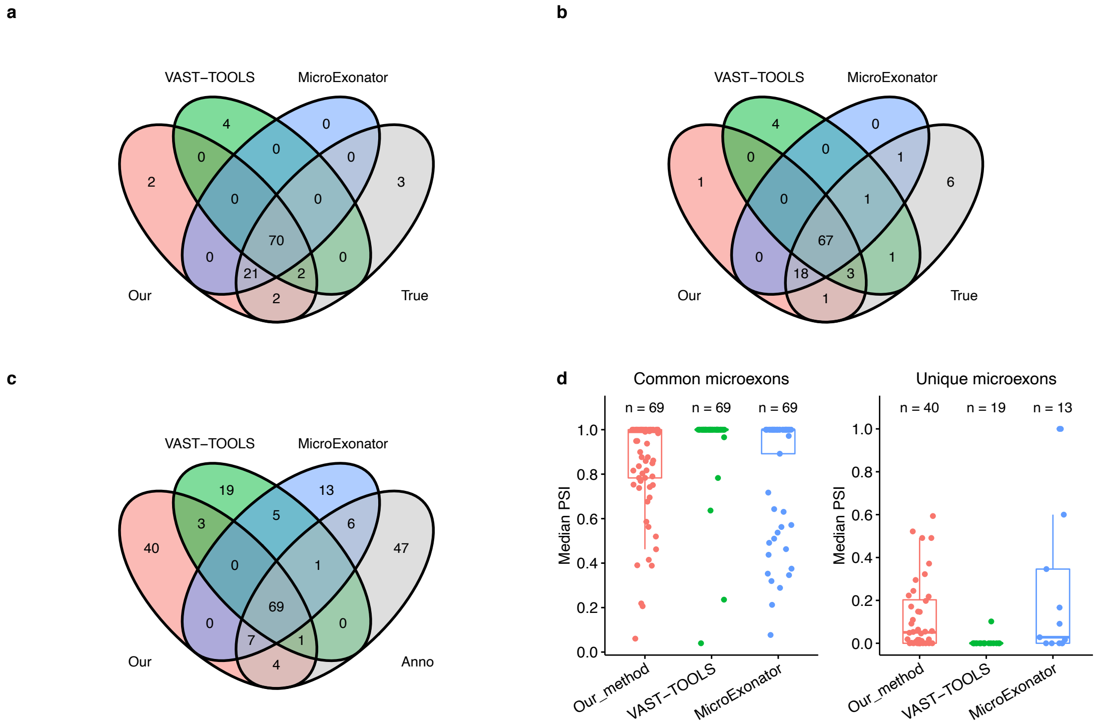

Supplementary Fig. 2: Comparison of MicroExonator, VAST-TOOLS, and our method for microexon discovery. **a**, The result from using simulated 2 × 100 bp paired-end read data. **b**, The result from using simulated 1 × 50 bp single-end read data. **c**, the result from using the real RNA-seq data. **d**, Microexon-inclusion levels of common and unique microexons from the real RNA-seq data by three different methods. PSI, percent spliced in. True, true microexons in simulation. Anno, annotated microexons from Araport11. For the box plots, the bounds of a box show the interquartile range (IQR), the center line in the box shows the median, and the whiskers extend to no further than 1.5 × IQR from the box bounds.

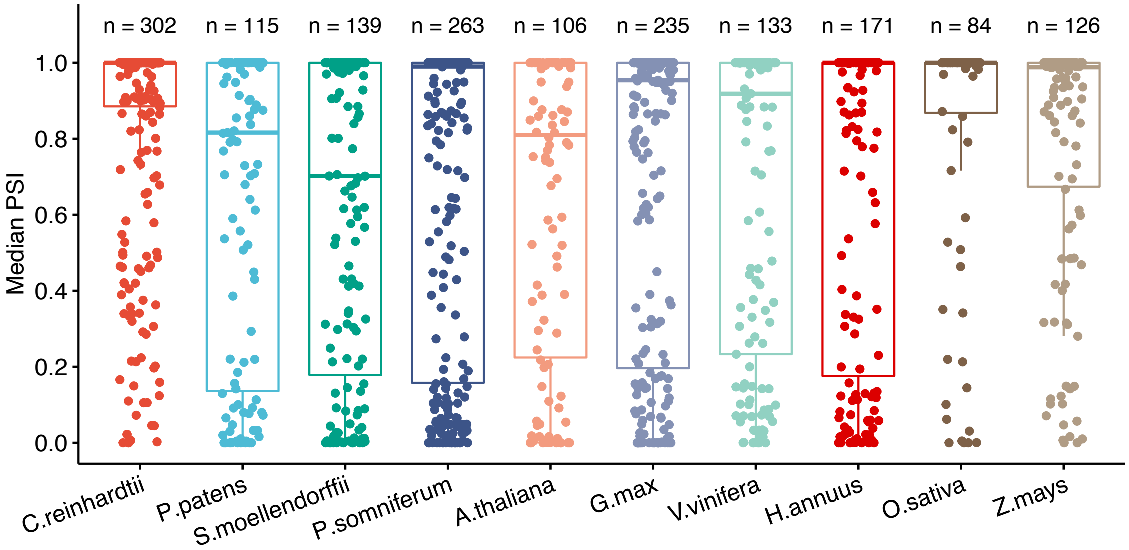

Supplementary Fig. 3: Distribution of coding microexon inclusion levels in 10 plant species. Microexons were discovered by our method. PSI, percent spliced in. Each dot is the median PSI value of one microexon in the population. For the box plots, the bounds of a box show the interquartile range (IQR), the center line in the box shows the median, and the whiskers extend to no further than 1.5 × IQR from the box bounds.

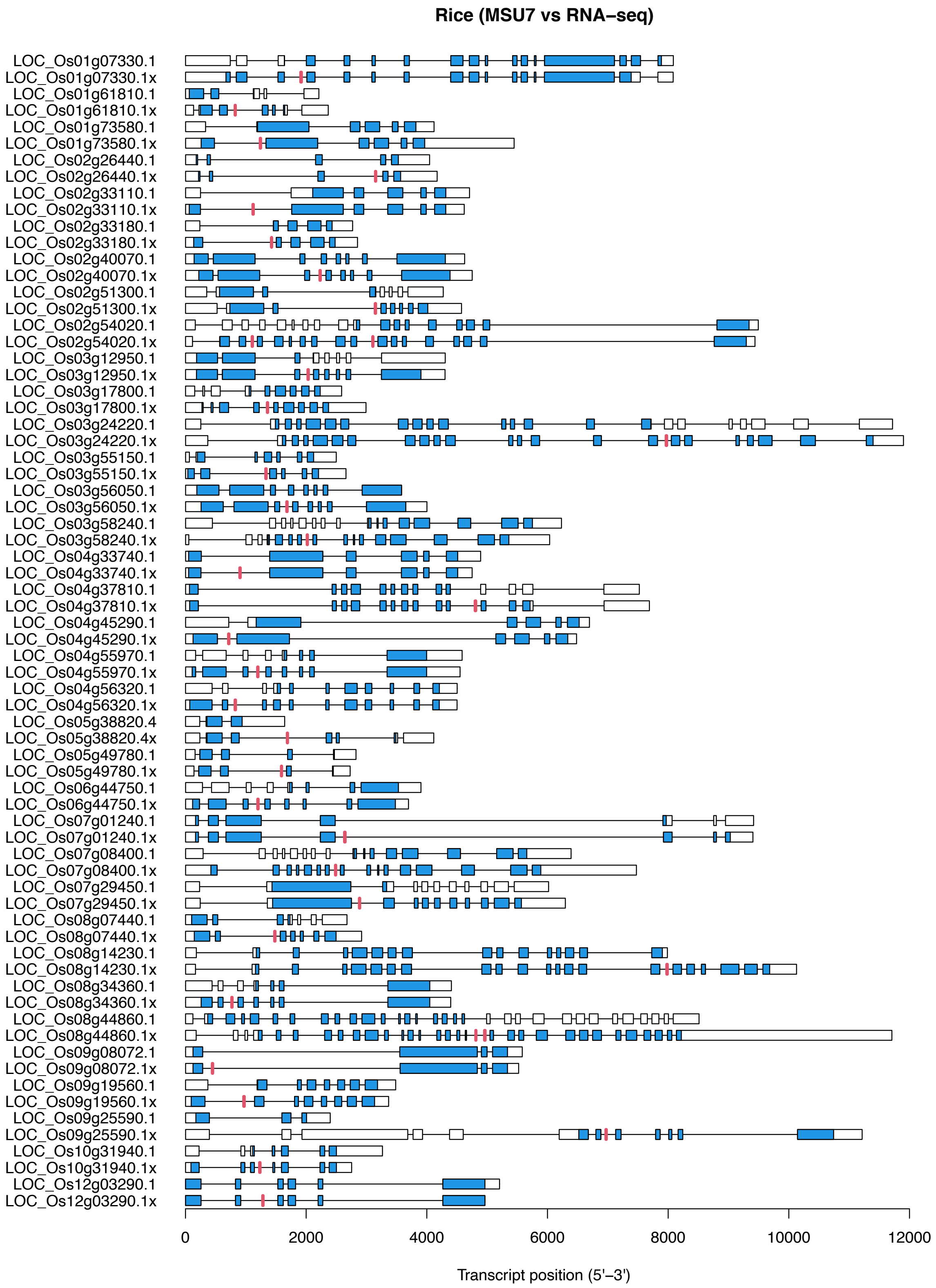

Supplementary Fig. 4: Pairwise comparison of RNA-seq assembled gene models containing microexons and reference gene models without microexons in rice. Transcripts ending with an "x" are assembled gene models from RNA-seq data and the others are from reference annotations (MSU7). Boxes are exons and segments connecting boxes are introns. Blue boxes indicate coding exons and white are untranslated regions (UTRs). Red small boxes are microexons.

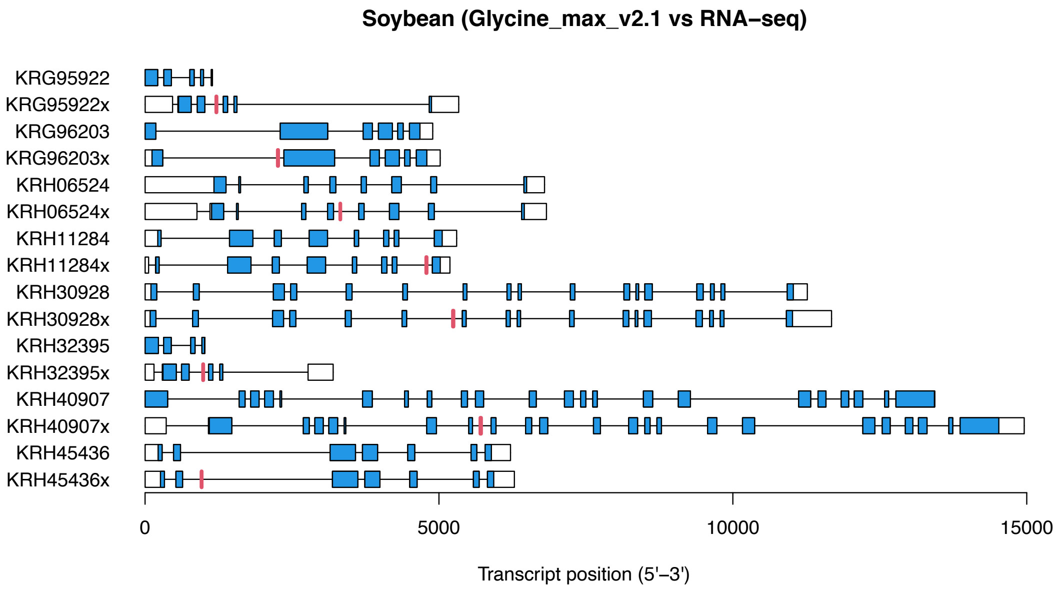

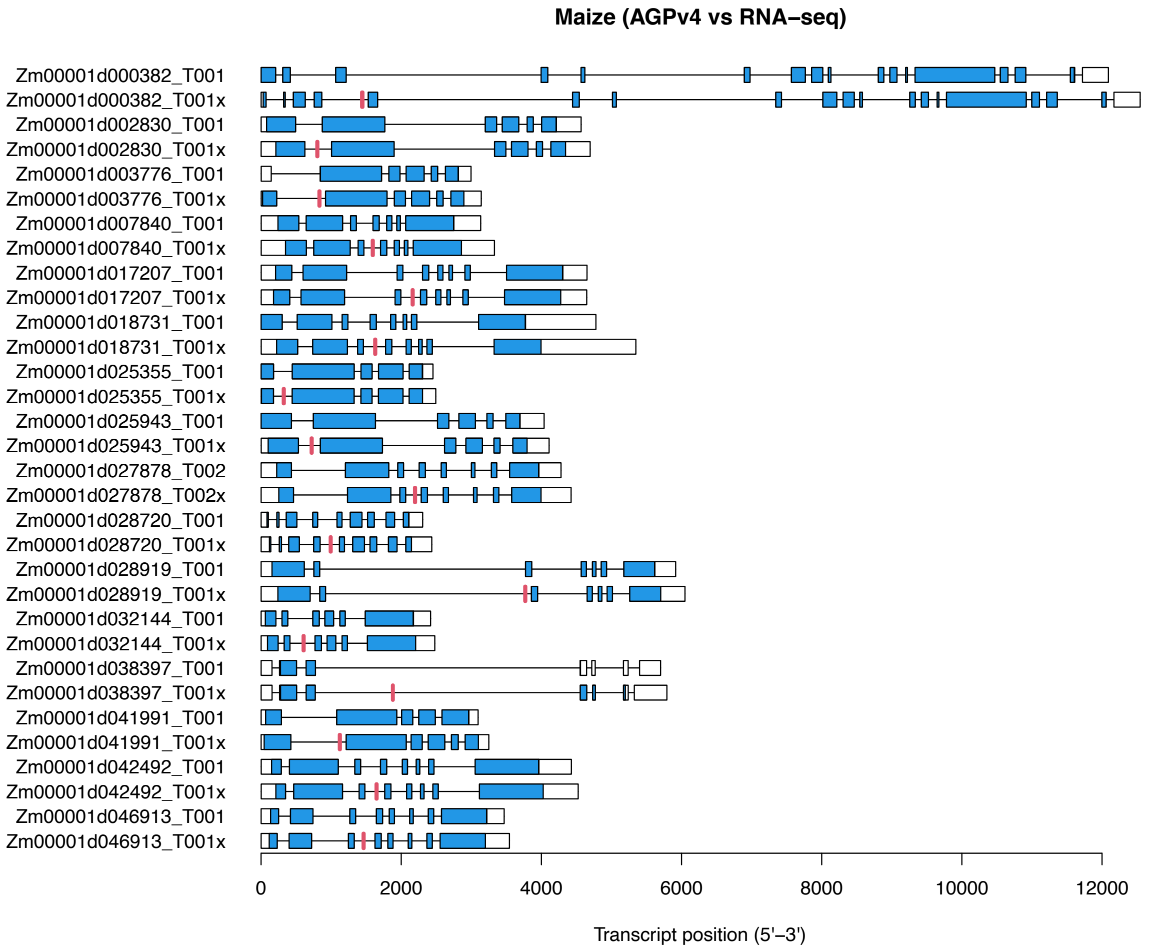

Supplementary Fig. 5: Pairwise comparison of RNA-seq assembled gene models containing microexons and reference gene models without microexons in soybean and maize. Only the transcripts spanning genomic regions less than 20 kb are shown. Transcripts ending with an “x” are assembled gene models from RNA-seq data and the others are from reference annotations. Boxes are exons and segments connecting boxes are introns.Blue boxes indicate coding exons and white are untranslated regions (UTRs). Red small boxes are microexons.

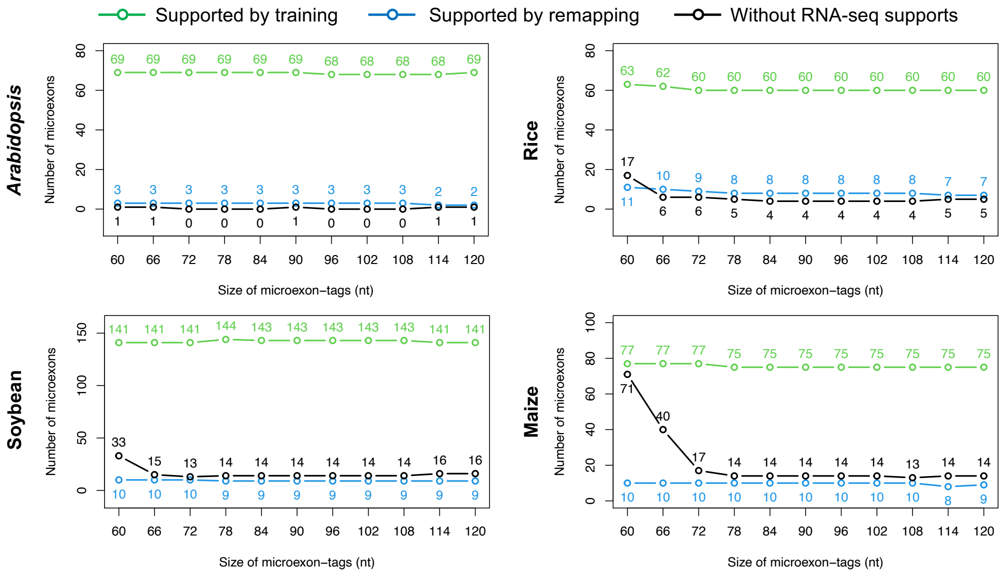

Supplementary Fig. 6: Effect of microexon-tag size on microexon modeling. The length of 108 nt was used because of its higher accuracy and lower false positives. In addition, because one microexon-tag may have multiple exons around the microexon, the size of 108 nt for microexon-tags makes two boundary exons have long enough size, i.e., in all 45 clusters, the minimal size of boundary exon parts included in the microexon-tag > 10bp, which can increase the searching accuracy and speed in prediction for very large genomes.

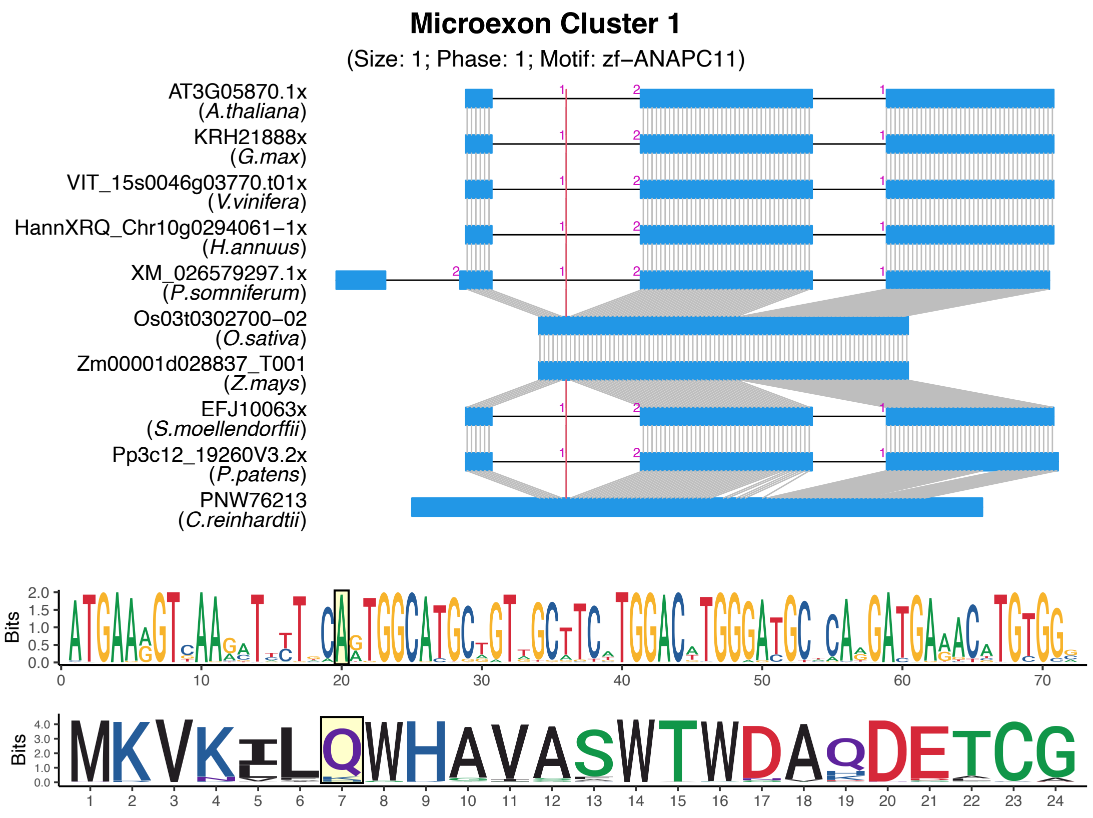

Supplementary Fig. 7: Multiple sequence alignments of microexon-containing genes and the homologs in 10 plant species, and DNA and AA sequence logos in microexon-tag Cluster 1.

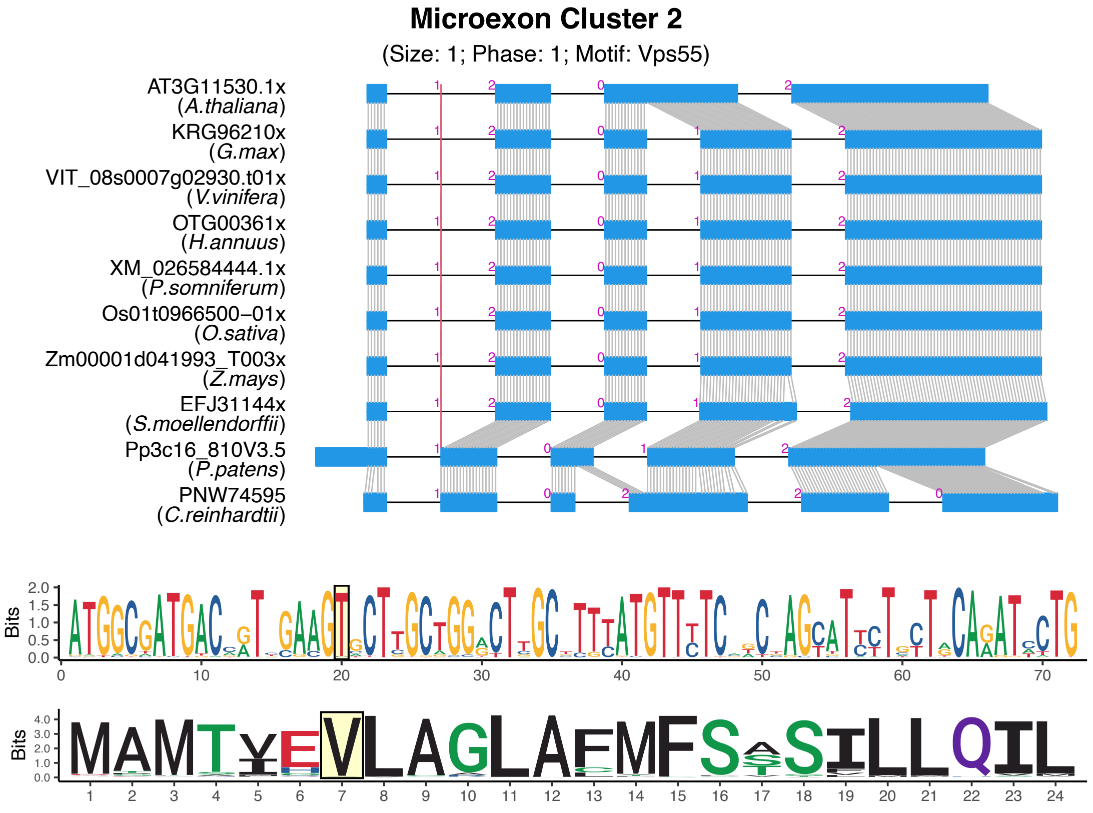

Supplementary Fig. 8: Multiple sequence alignments of microexon-containing genes and the homologs in 10 plant species, and DNA and AA sequence logos in microexon-tag Cluster 2.

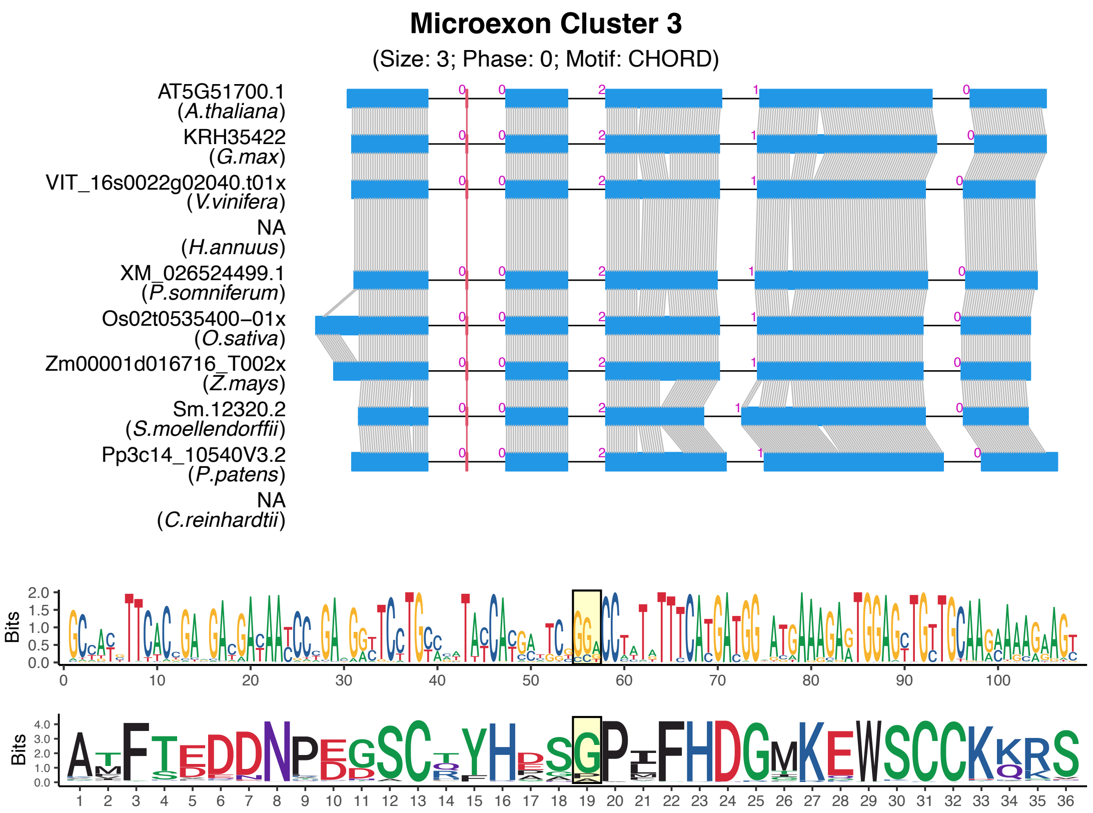

Supplementary Fig. 9: Multiple sequence alignments of microexon-containing genes and the homologs in 10 plant species, and DNA and AA sequence logos in microexon-tag Cluster 3.

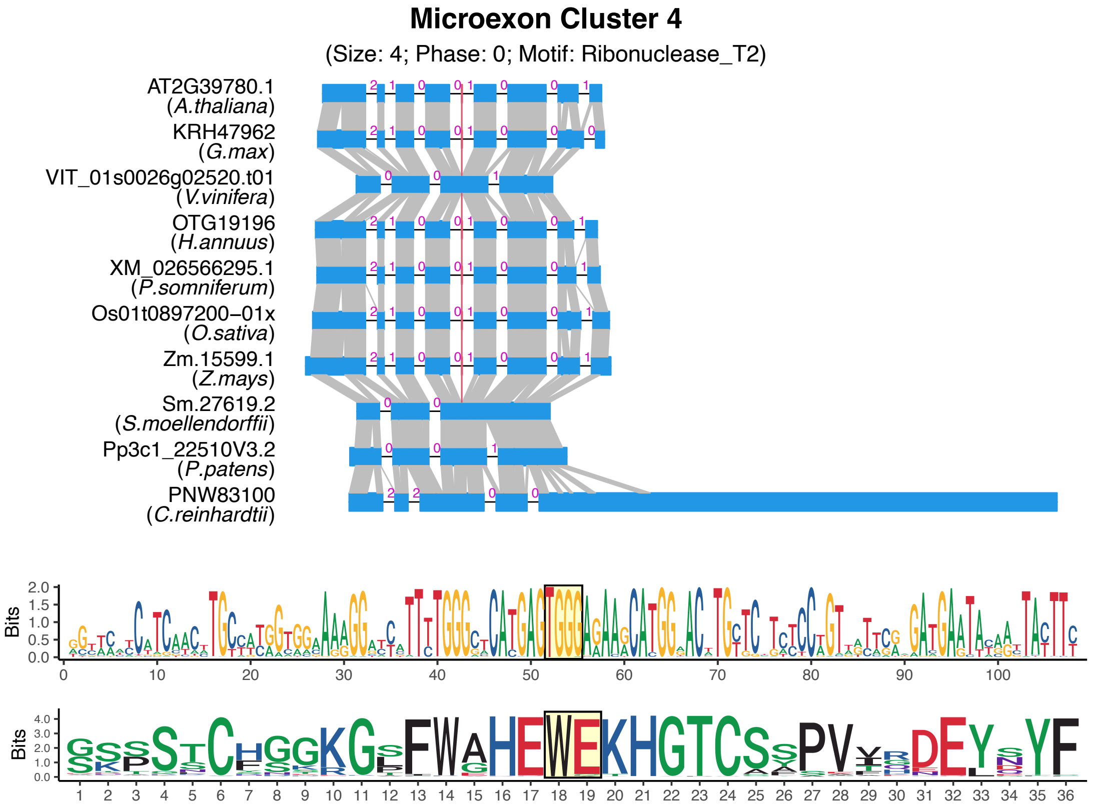

Supplementary Fig. 10: Multiple sequence alignments of microexon-containing genes and the homologs in 10 plant species, and DNA and AA sequence logos in microexon-tag Cluster 4.

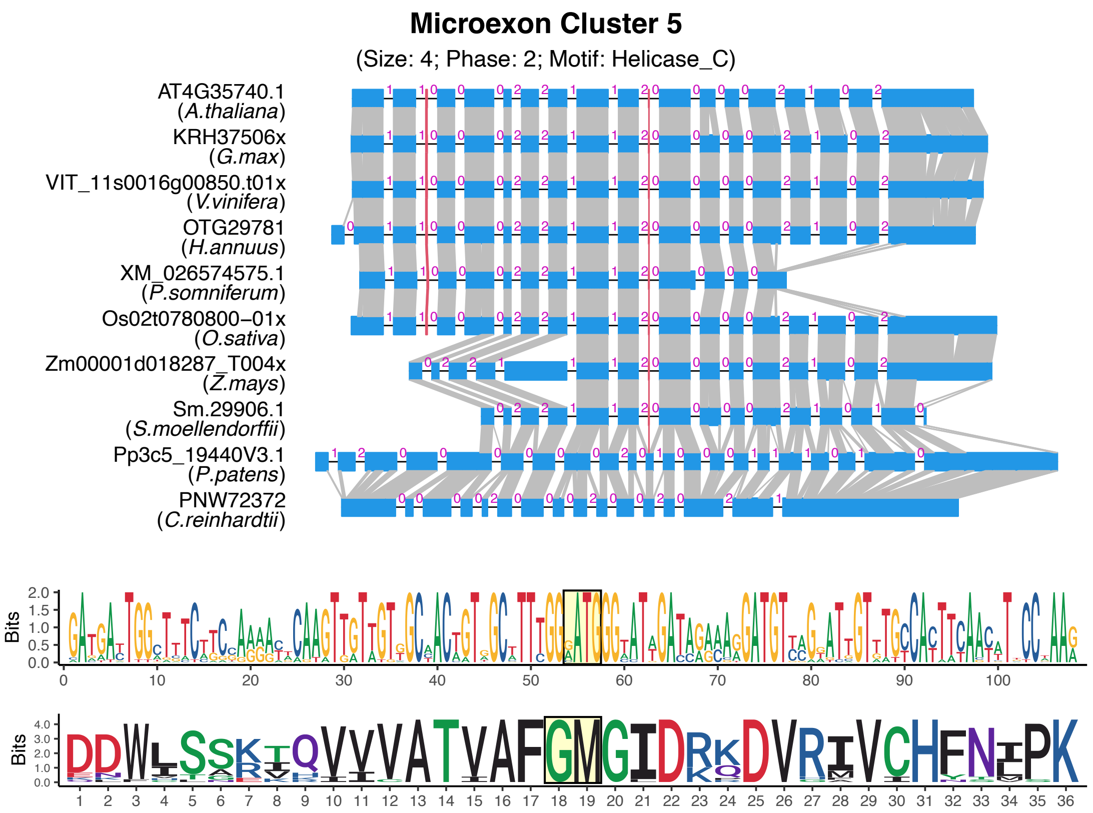

Supplementary Fig. 11: Multiple sequence alignments of microexon-containing genes and the homologs in 10 plant species, and DNA and AA sequence logos in microexon-tag Cluster 5.

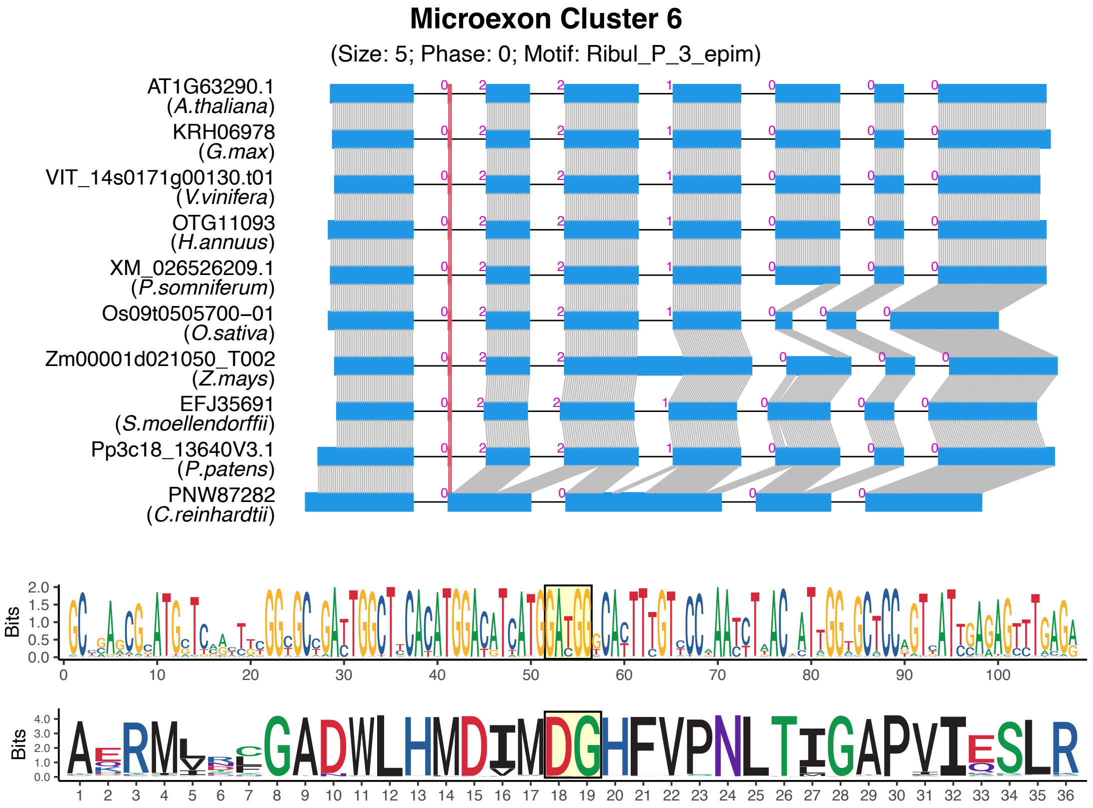

Supplementary Fig. 12: Multiple sequence alignments of microexon-containing genes and the homologs in 10 plant species, and DNA and AA sequence logos in microexon-tag Cluster 6.

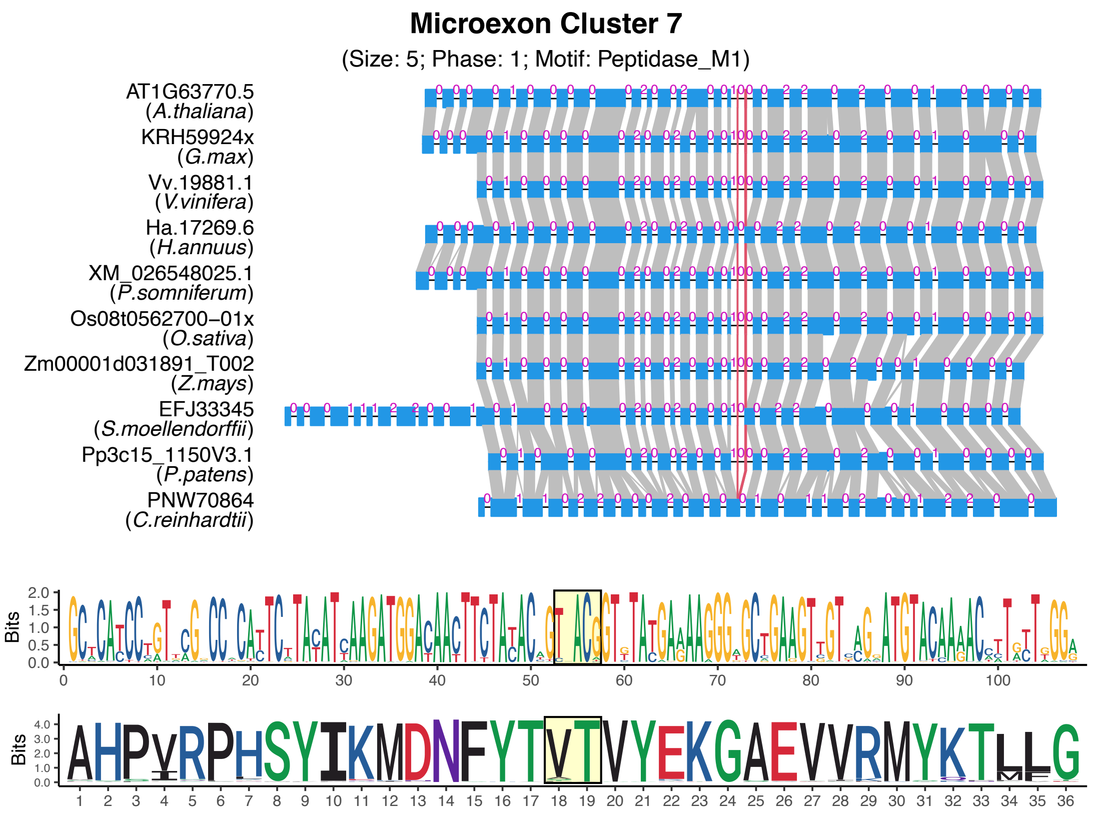

Supplementary Fig. 13: Multiple sequence alignments of microexon-containing genes and the homologs in 10 plant species, and DNA and AA sequence logos in microexon-tag Cluster 7.

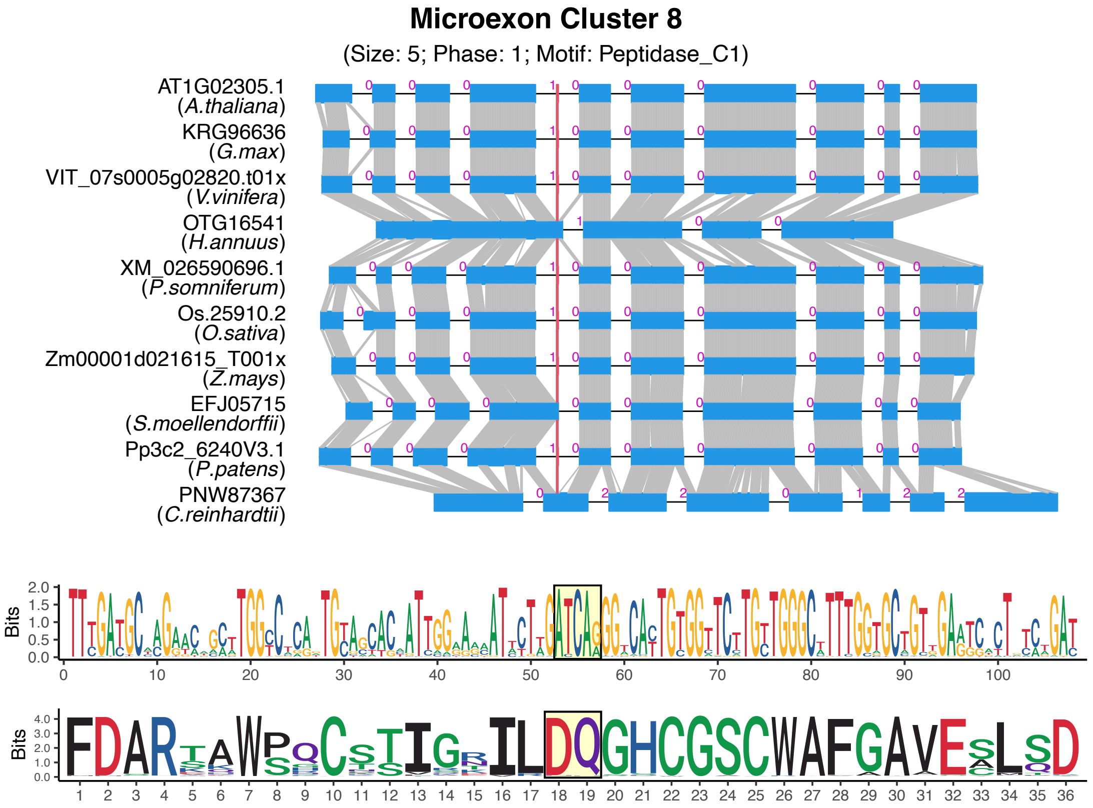

Supplementary Fig. 14: Multiple sequence alignments of microexon-containing genes and the homologs in 10 plant species, and DNA and AA sequence logos in microexon-tag Cluster 8.

Microexon Cluster 9

(Size: 5; Phase: 2; Motif: SKG6)

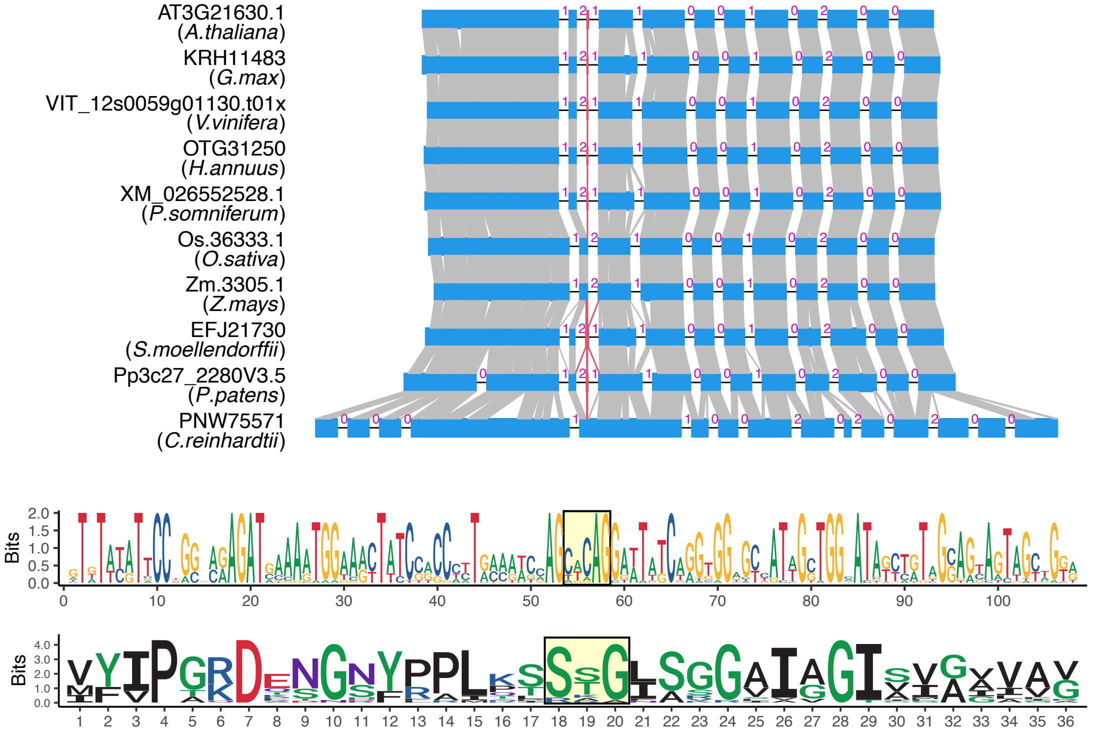

Supplementary Fig. 15: Multiple sequence alignments of microexon-containing genes and the homologs in 10 plant species, and DNA and AA sequence logos in microexon-tag Cluster 9.

Microexon Cluster 10

(Size: 6; Phase: 0; Motif: DUF4788)

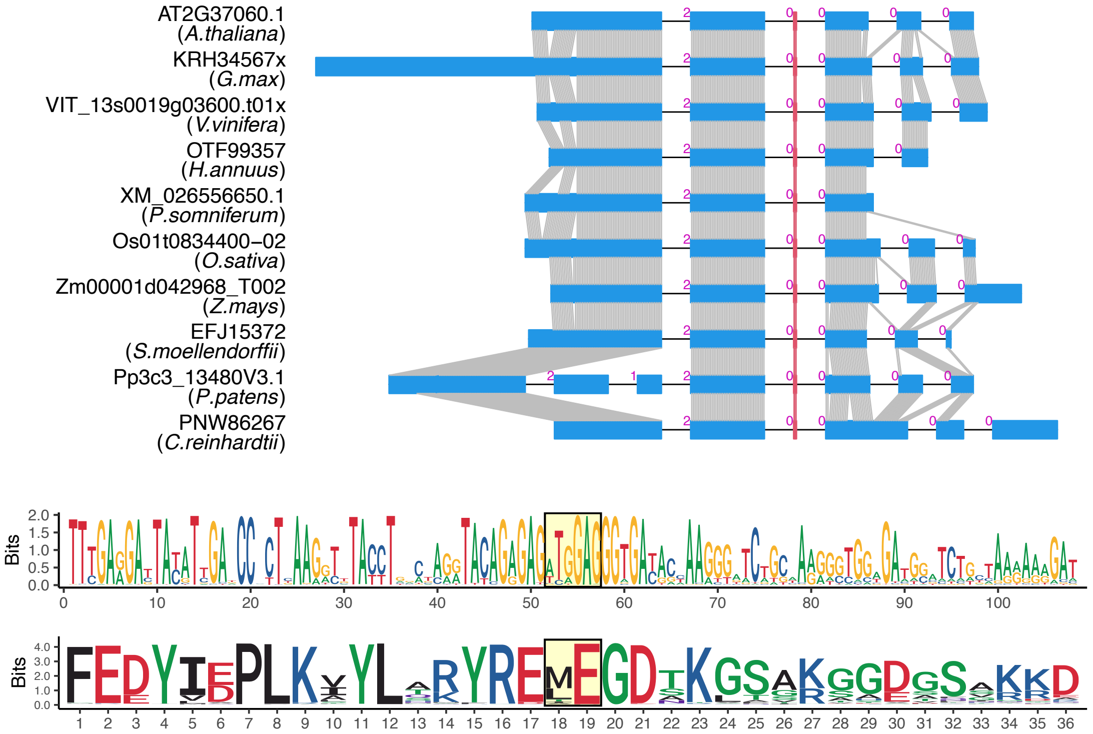

Supplementary Fig. 16: Multiple sequence alignments of microexon-containing genes and the homologs in 10 plant species, and DNA and AA sequence logos in microexon-tag Cluster 10.

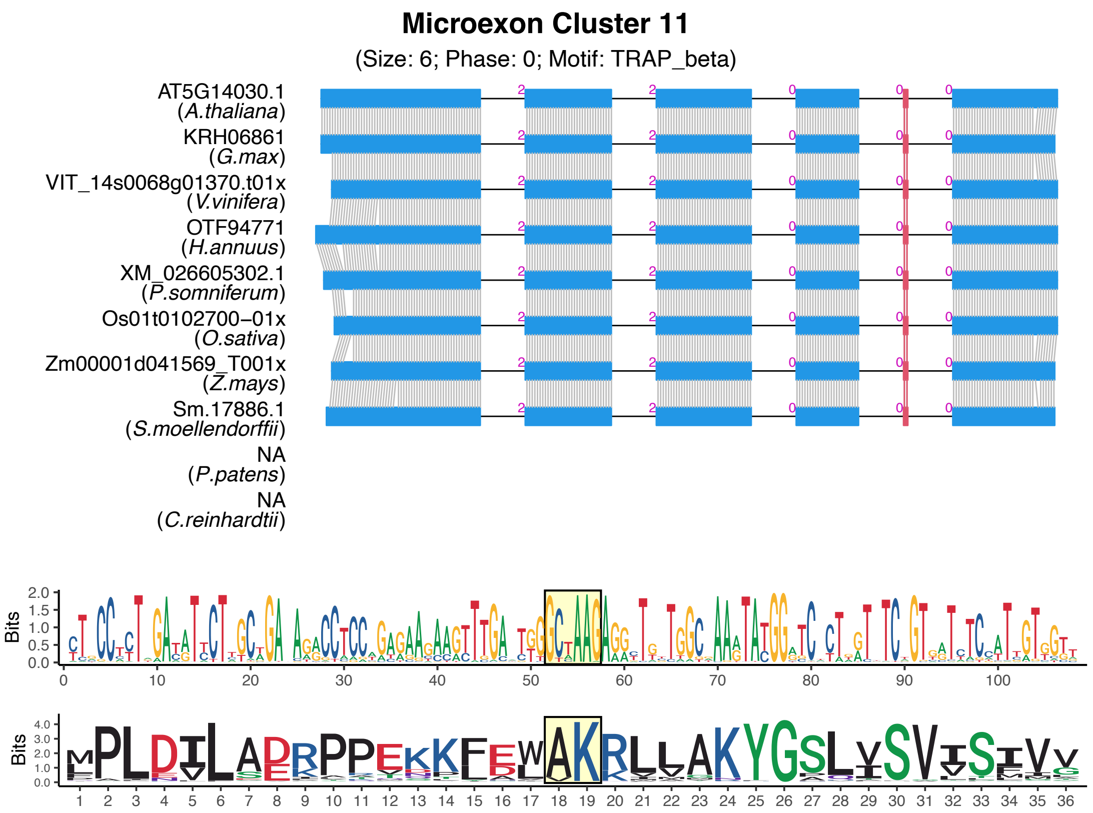

Supplementary Fig. 17: Multiple sequence alignments of microexon-containing genes and the homologs in 10 plant species, and DNA and AA sequence logos in microexon-tag Cluster 11.

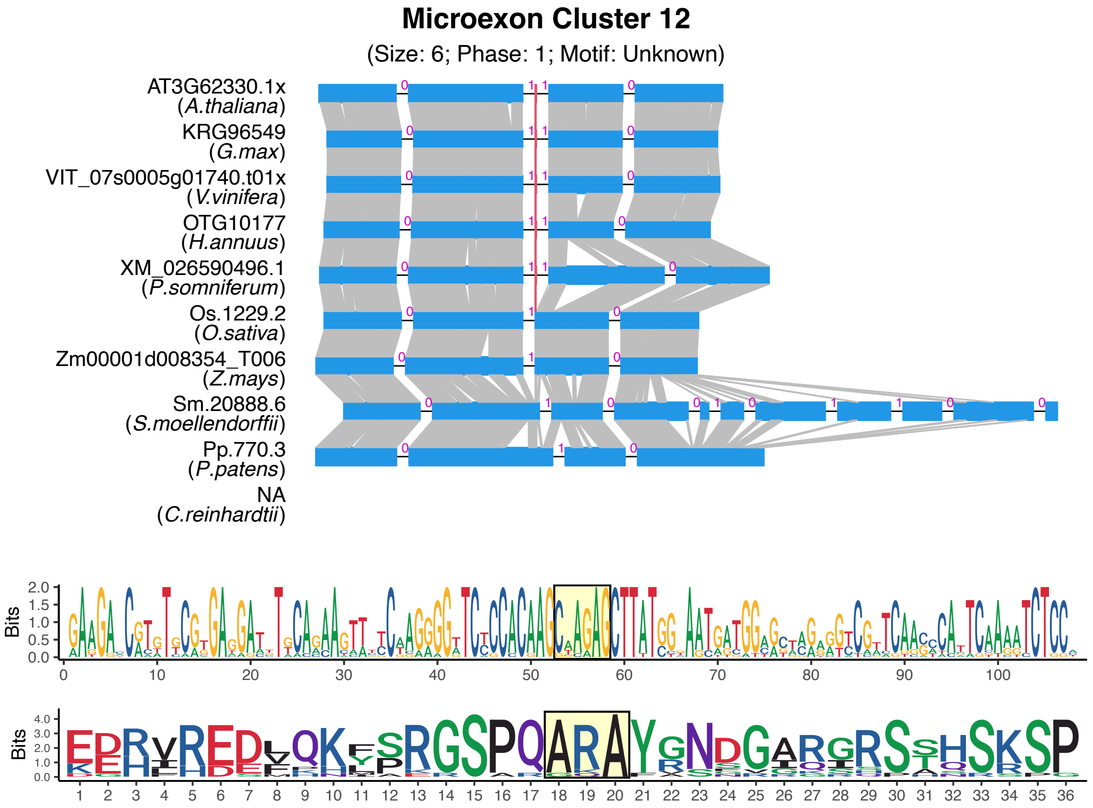

Supplementary Fig. 18: Multiple sequence alignments of microexon-containing genes and the homologs in 10 plant species, and DNA and AA sequence logos in microexon-tag Cluster 12.

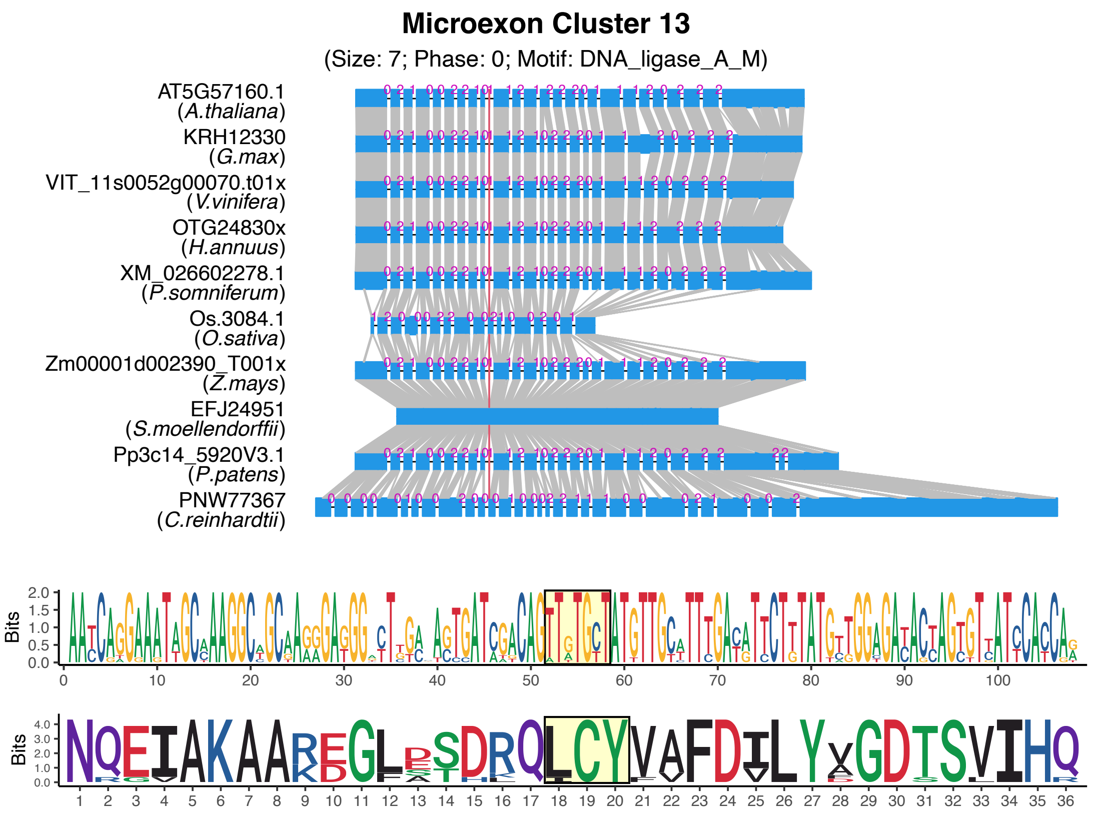

Supplementary Fig. 19: Multiple sequence alignments of microexon-containing genes and the homologs in 10 plant species, and DNA and AA sequence logos in microexon-tag Cluster 13.

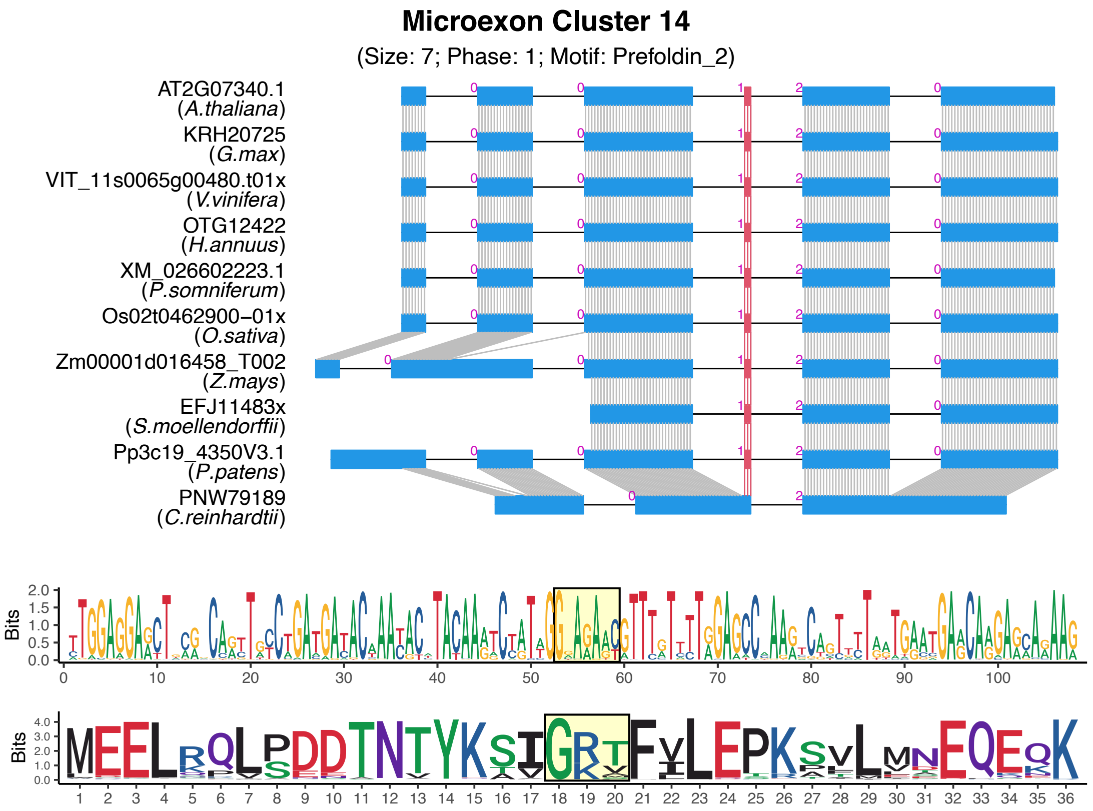

Supplementary Fig. 20: Multiple sequence alignments of microexon-containing genes and the homologs in 10 plant species, and DNA and AA sequence logos in microexon-tag Cluster 14.

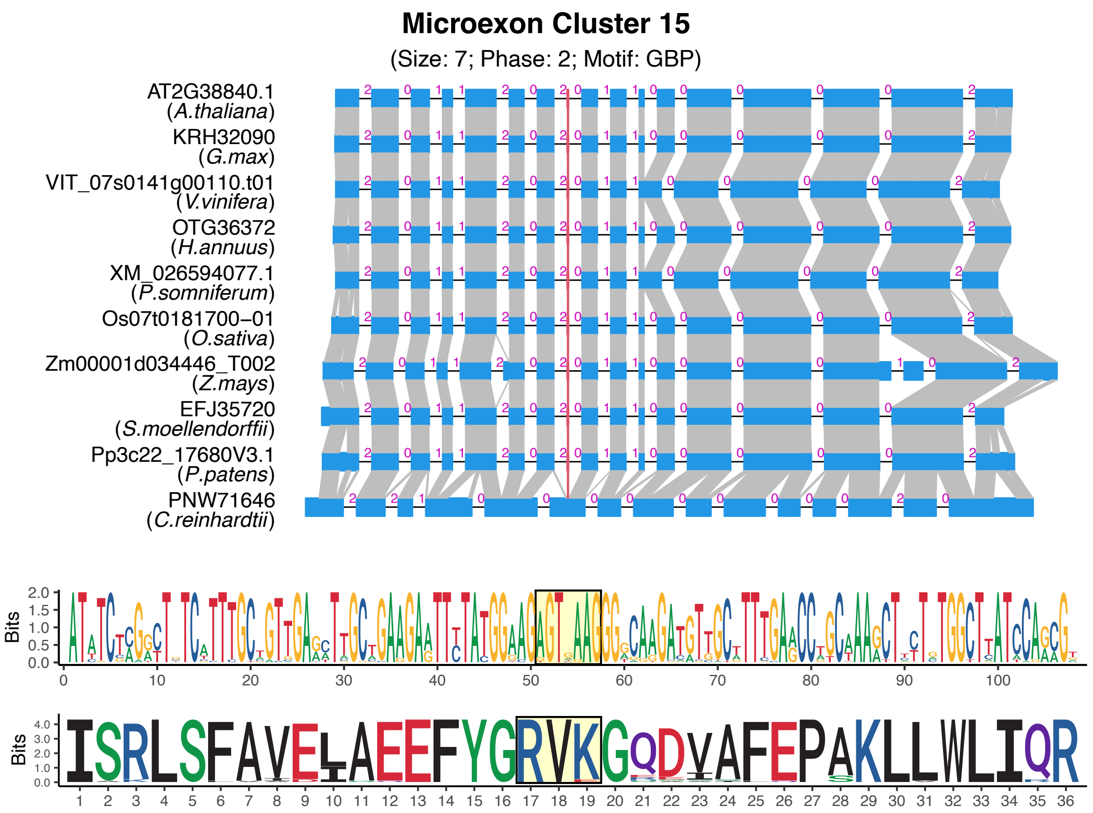

Supplementary Fig. 21: Multiple sequence alignments of microexon-containing genes and the homologs in 10 plant species, and DNA and AA sequence logos in microexon-tag Cluster 15.

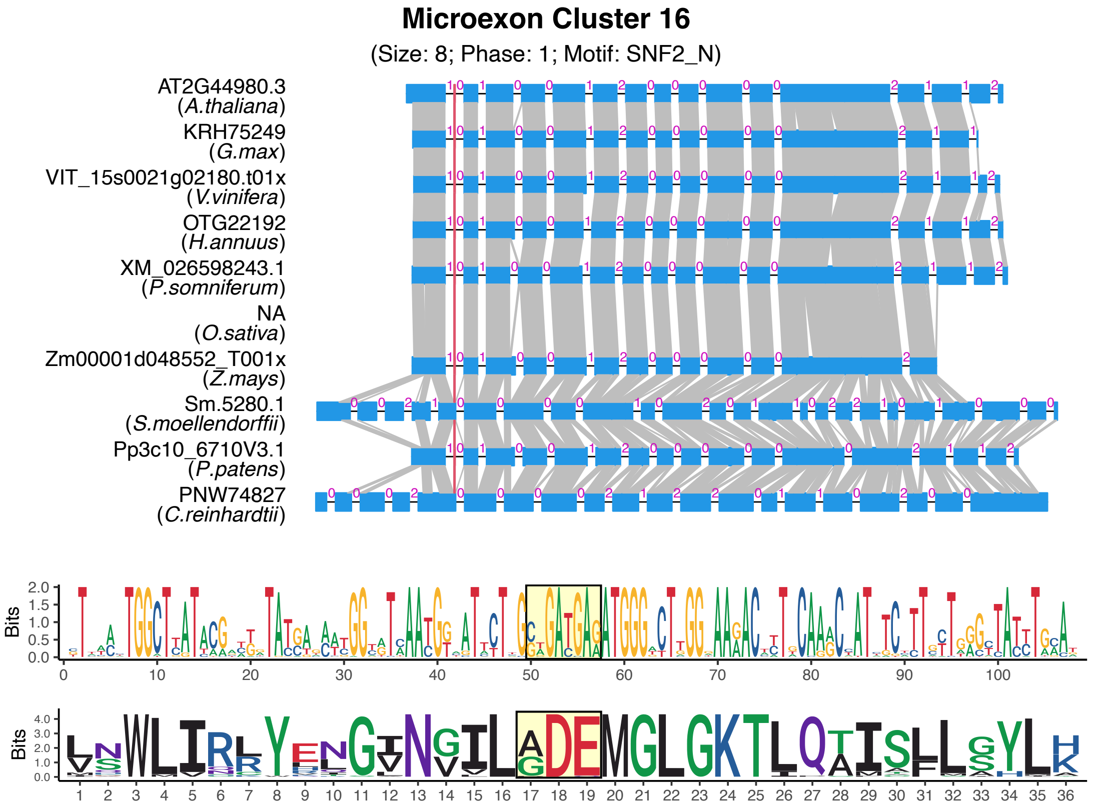

Supplementary Fig. 22: Multiple sequence alignments of microexon-containing genes and the homologs in 10 plant species, and DNA and AA sequence logos in microexon-tag Cluster 16.

Microexon Cluster 17

(Size: 8; Phase: 1; Motif: IP_trans)

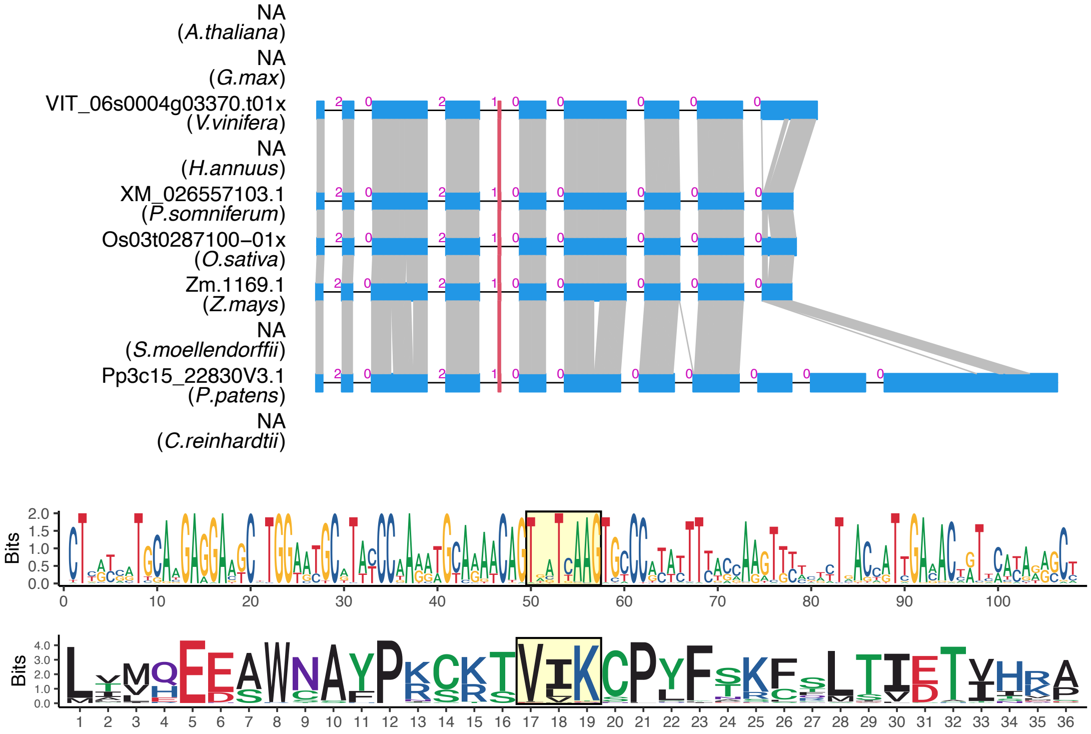

Supplementary Fig. 23: Multiple sequence alignments of microexon-containing genes and the homologs in 10 plant species, and DNA and AA sequence logos in microexon-tag Cluster 17.

Microexon Cluster 18

(Size: 8; Phase: 1; Motif: DEAD)

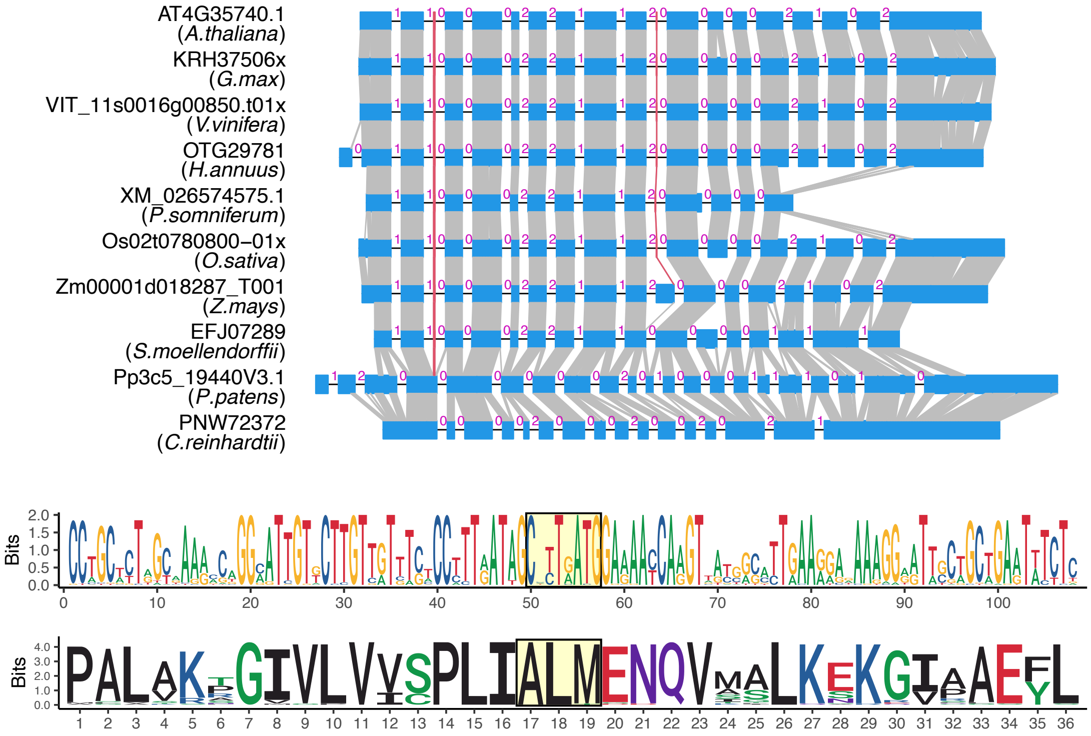

Supplementary Fig. 24: Multiple sequence alignments of microexon-containing genes and the homologs in 10 plant species, and DNA and AA sequence logos in microexon-tag Cluster 18.

Microexon Cluster 19

(Size: 8; Phase: 2; Motif: Unknown)

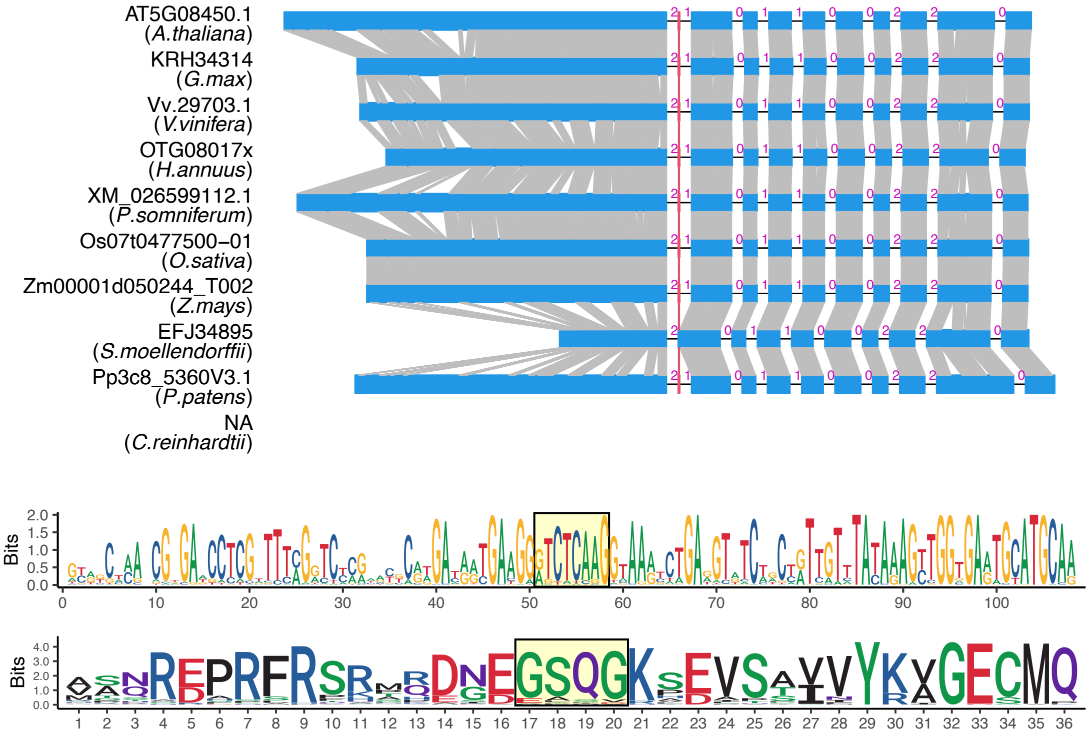

Supplementary Fig. 25: Multiple sequence alignments of microexon-containing genes and the homologs in 10 plant species, and DNA and AA sequence logos in microexon-tag Cluster 19.

Microexon Cluster 20

(Size: 8; Phase: 2; Motif: VSP)

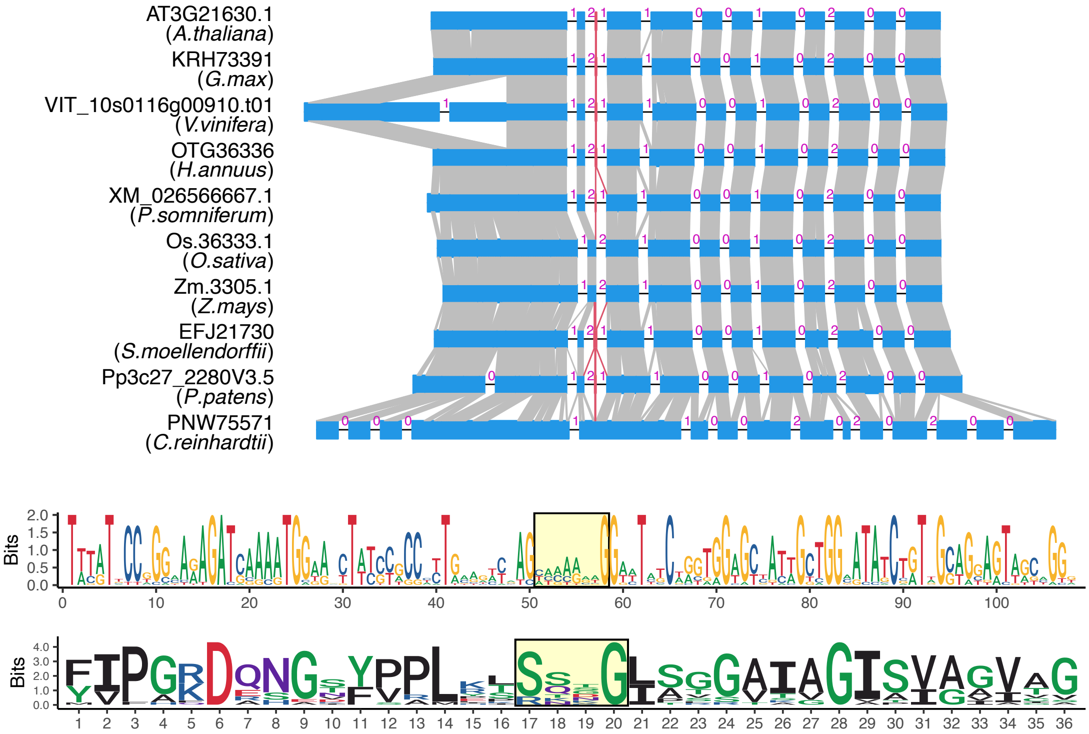

Supplementary Fig. 26: Multiple sequence alignments of microexon-containing genes and the homologs in 10 plant species, and DNA and AA sequence logos in microexon-tag Cluster 20.

Microexon Cluster 21

(Size: 9; Phase: 1; Motif: AP2)

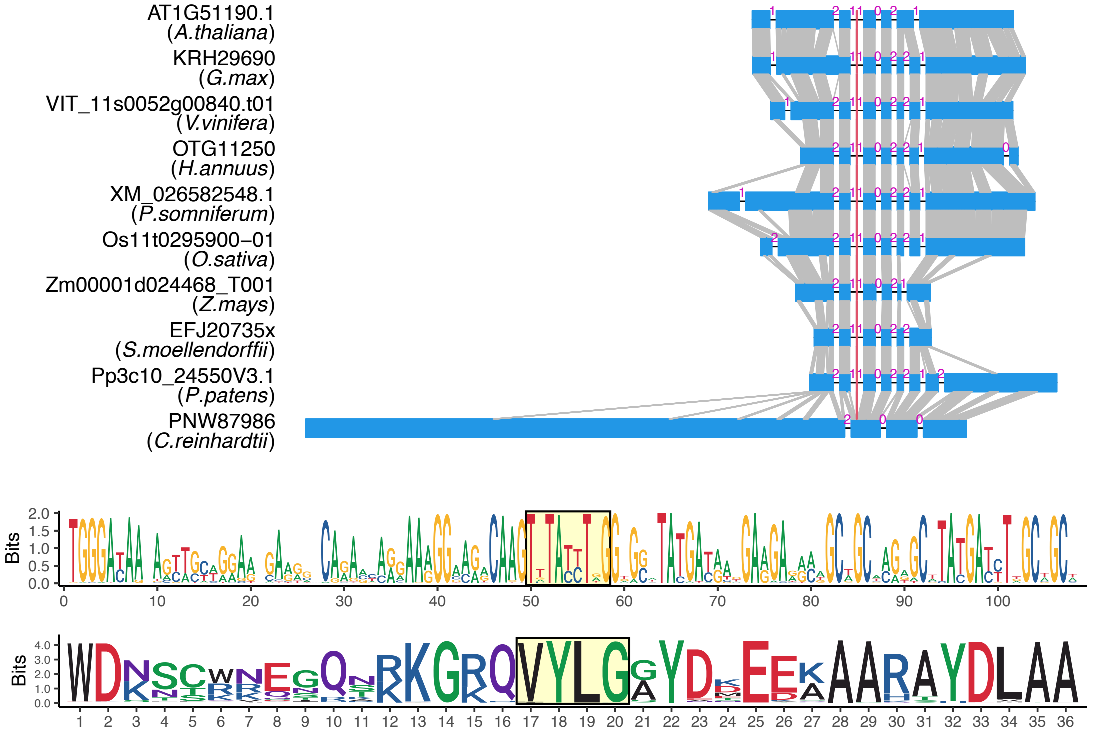

Supplementary Fig. 27: Multiple sequence alignments of microexon-containing genes and the homologs in 10 plant species, and DNA and AA sequence logos in microexon-tag Cluster 21.

Microexon Cluster 22

(Size: 9; Phase: 1; Motif: Glyco_hydro_32N)

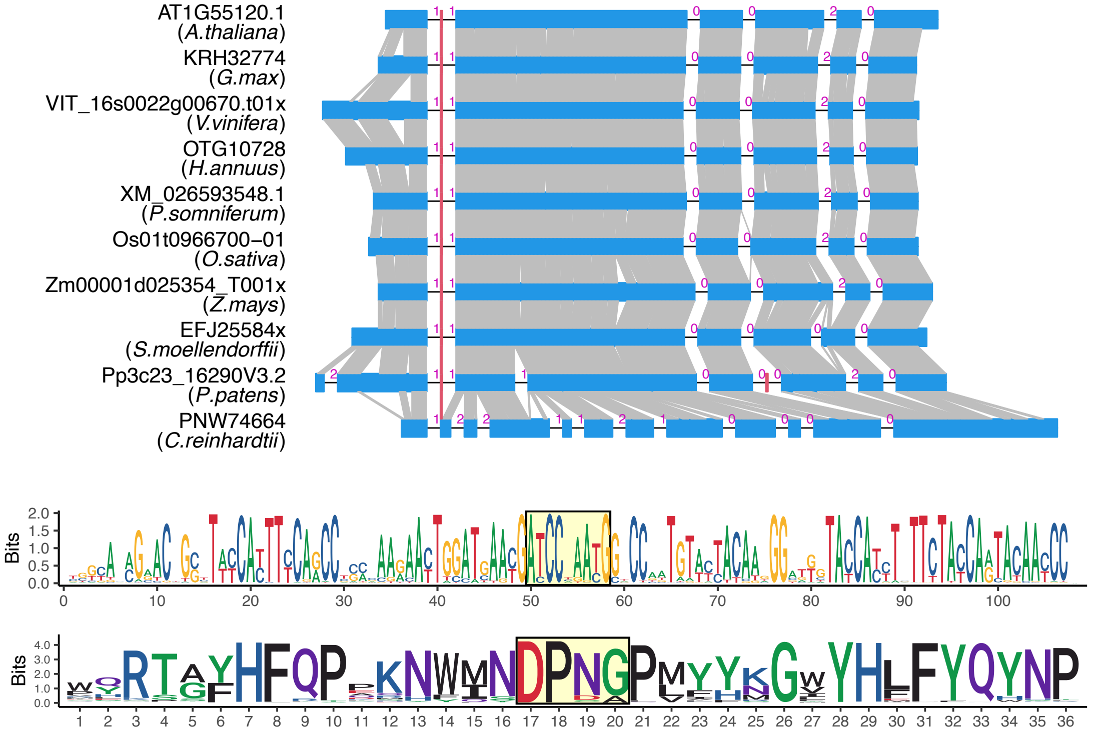

Supplementary Fig. 28: Multiple sequence alignments of microexon-containing genes and the homologs in 10 plant species, and DNA and AA sequence logos in microexon-tag Cluster 22.

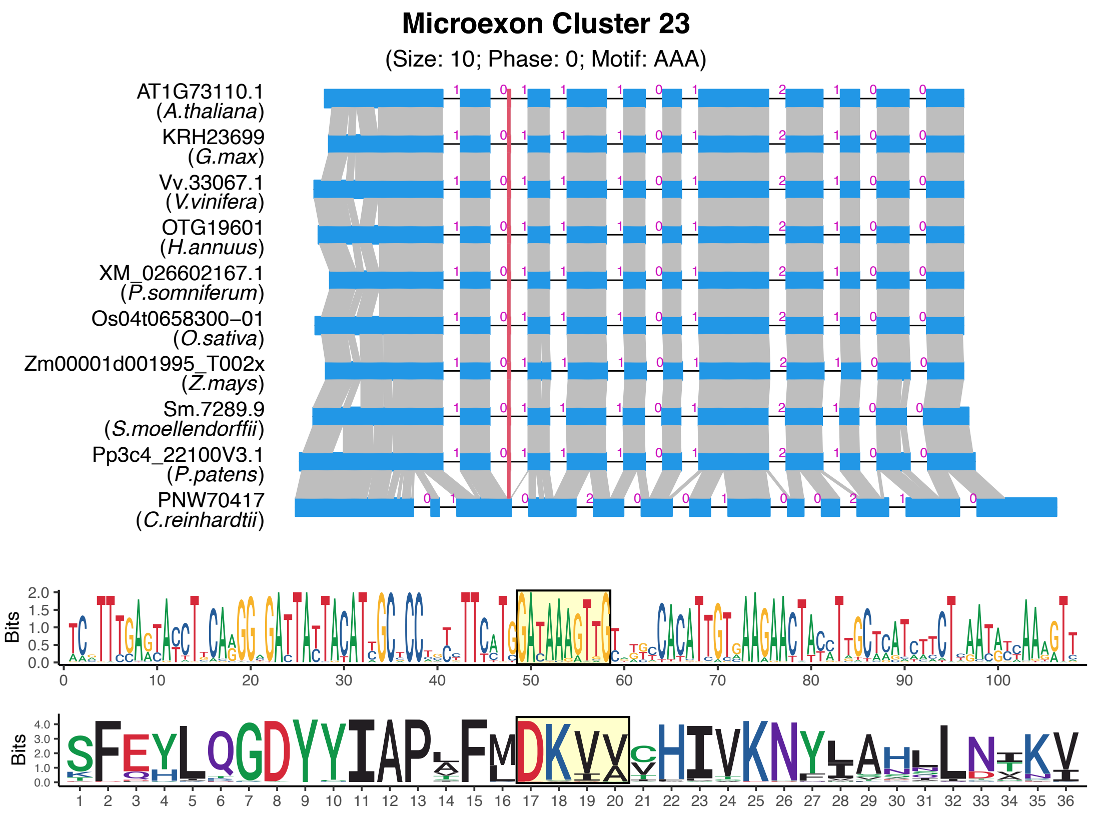

Supplementary Fig. 29: Multiple sequence alignments of microexon-containing genes and the homologs in 10 plant species, and DNA and AA sequence logos in microexon-tag Cluster 23.

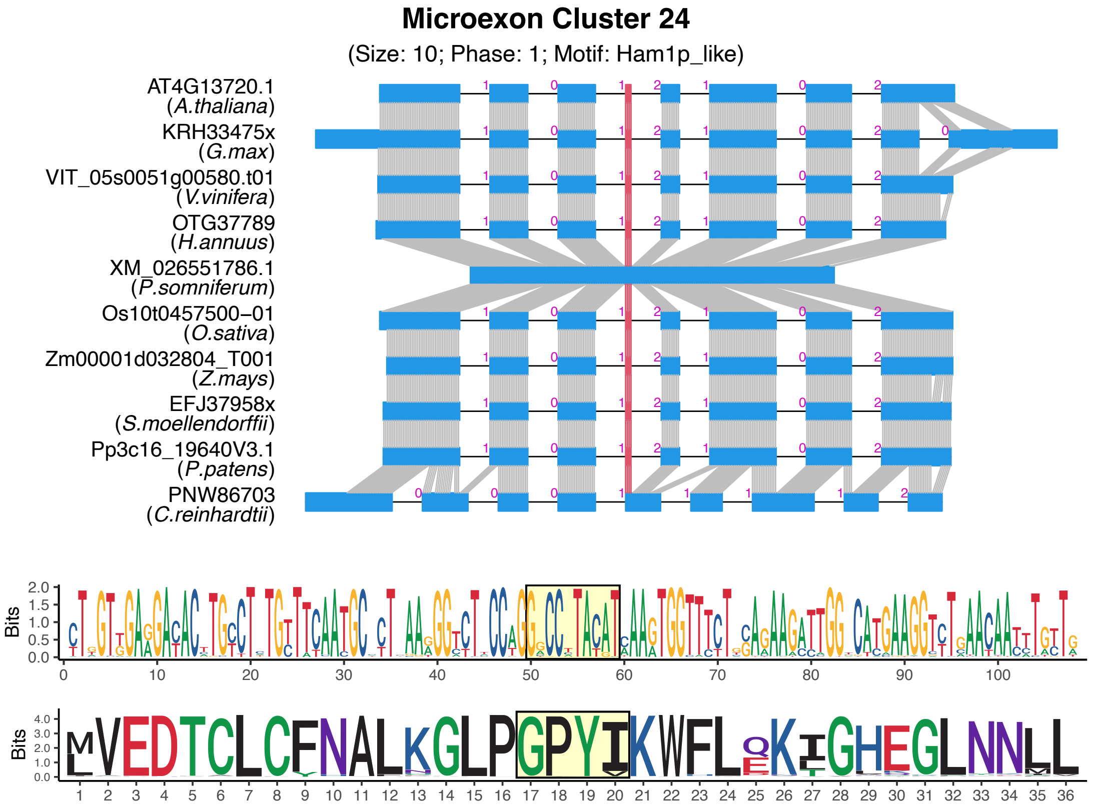

Supplementary Fig. 30: Multiple sequence alignments of microexon-containing genes and the homologs in 10 plant species, and DNA and AA sequence logos in microexon-tag Cluster 24.

Microexon Cluster 25

(Size: 10; Phase: 2; Motif: CDP−OH_P_transf)

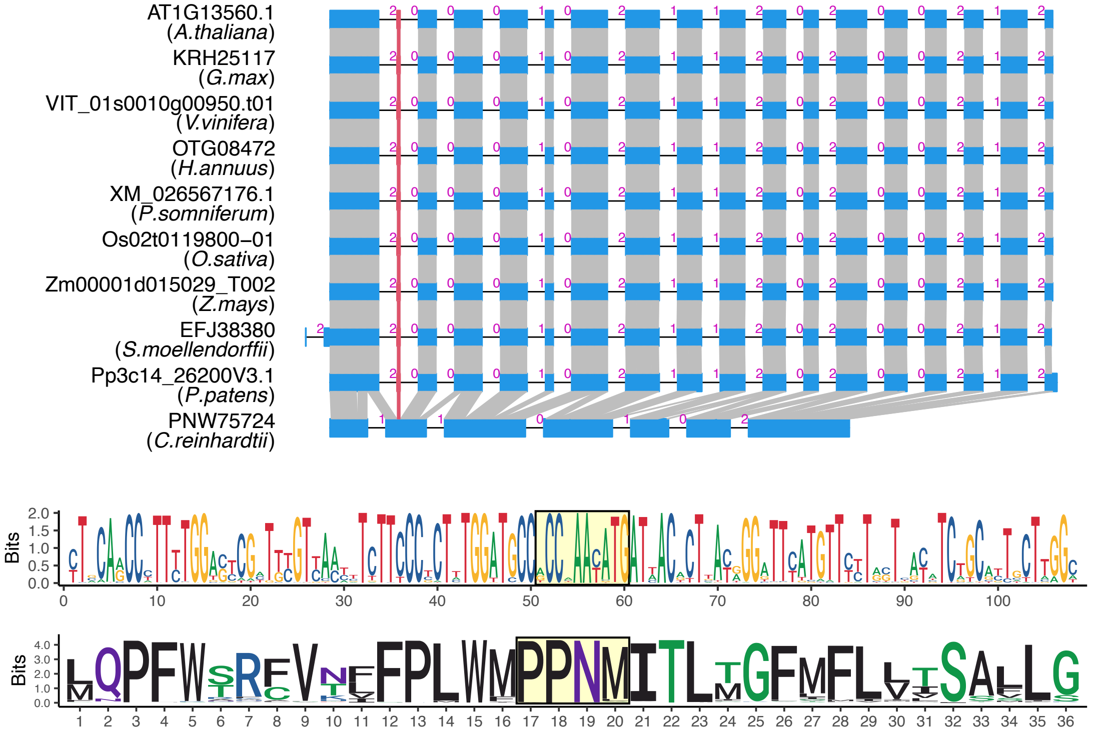

Supplementary Fig. 31: Multiple sequence alignments of microexon-containing genes and the homologs in 10 plant species, and DNA and AA sequence logos in microexon-tag Cluster 25.

Microexon Cluster 26

(Size: 11; Phase: 1; Motif: PRMT5)

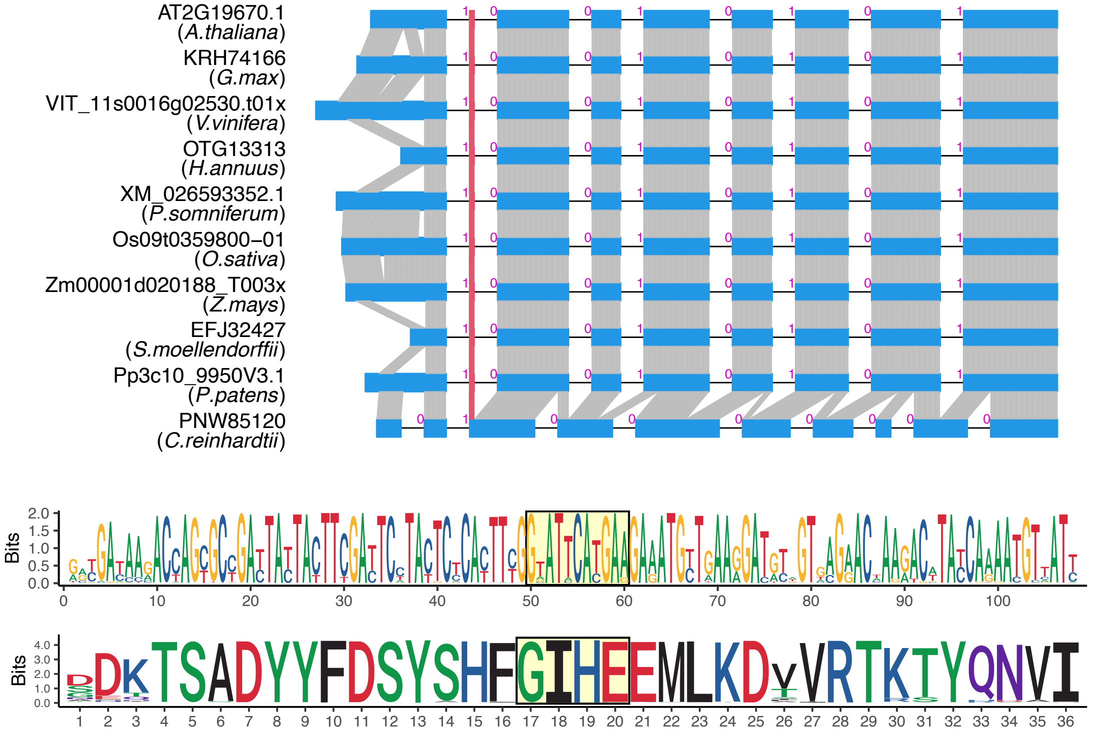

Supplementary Fig. 32: Multiple sequence alignments of microexon-containing genes and the homologs in 10 plant species, and DNA and AA sequence logos in microexon-tag Cluster 26.

Microexon Cluster 27

(Size: 11; Phase: 1; Motif: Gelsolin)

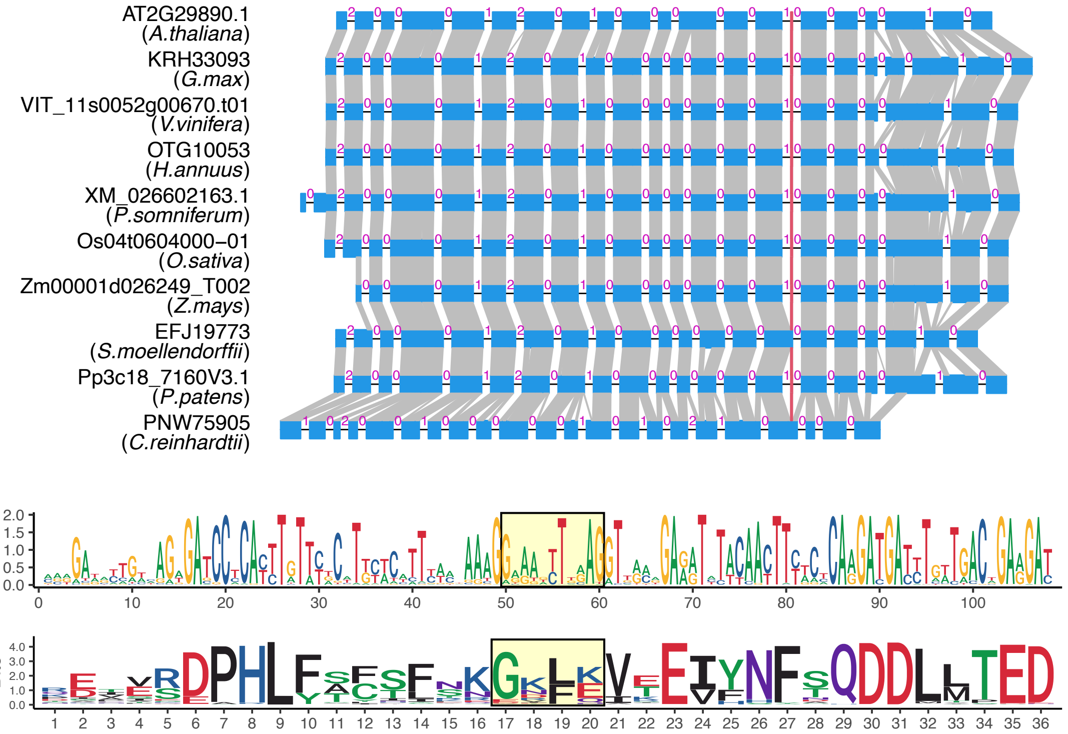

Supplementary Fig. 33: Multiple sequence alignments of microexon-containing genes and the homologs in 10 plant species, and DNA and AA sequence logos in microexon-tag Cluster 27.

Microexon Cluster 28

(Size: 12; Phase: 0; Motif: Peptidase_M1)

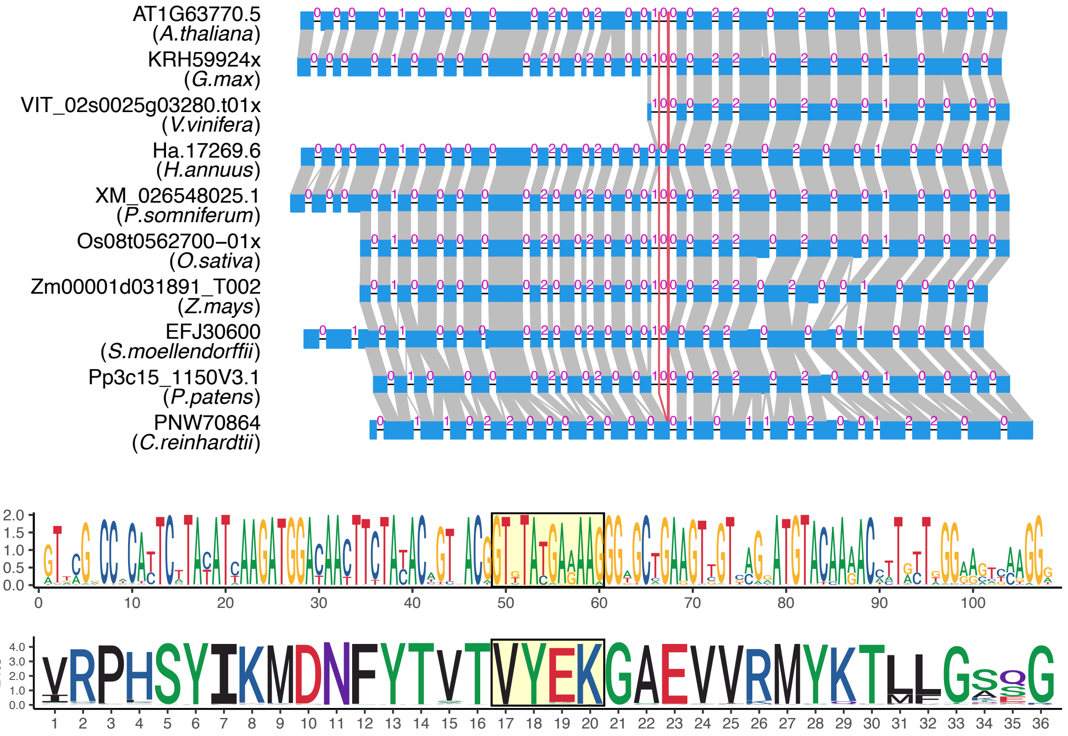

Supplementary Fig. 34: Multiple sequence alignments of microexon-containing genes and the homologs in 10 plant species, and DNA and AA sequence logos in microexon-tag Cluster 28.

Microexon Cluster 29

(Size: 12; Phase: 0; Motif: TPR_12)

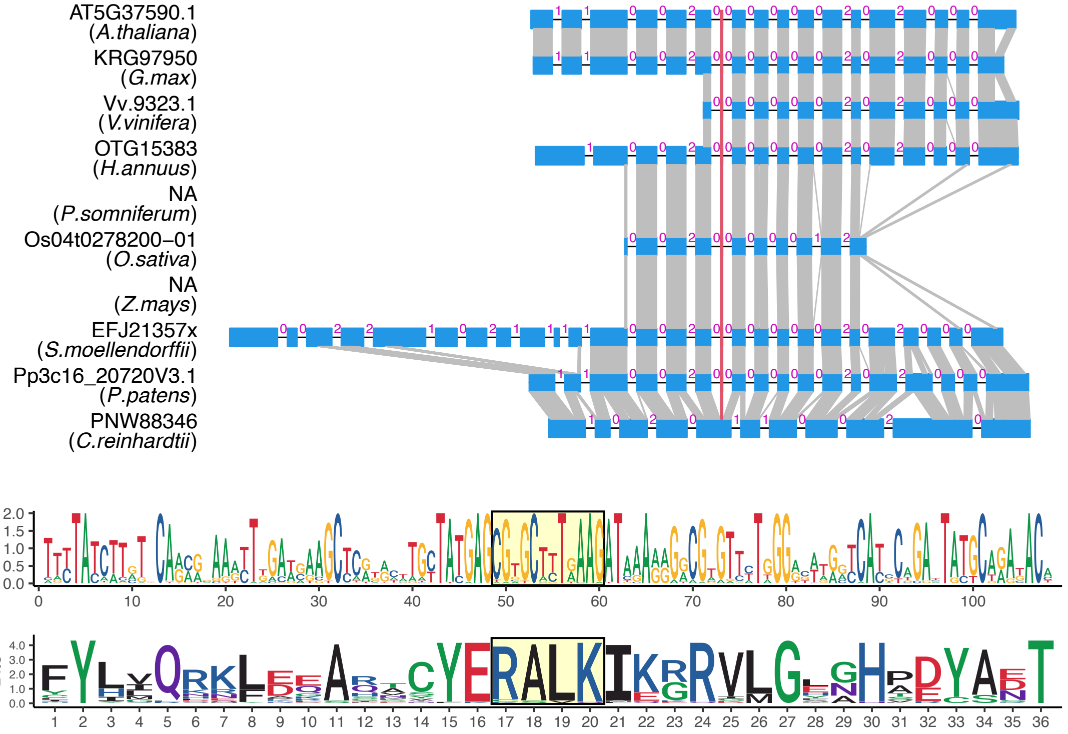

Supplementary Fig. 35: Multiple sequence alignments of microexon-containing genes and the homologs in 10 plant species, and DNA and AA sequence logos in microexon-tag Cluster 29.

Microexon Cluster 30

(Size: 12; Phase: 1; Motif: SPARK)

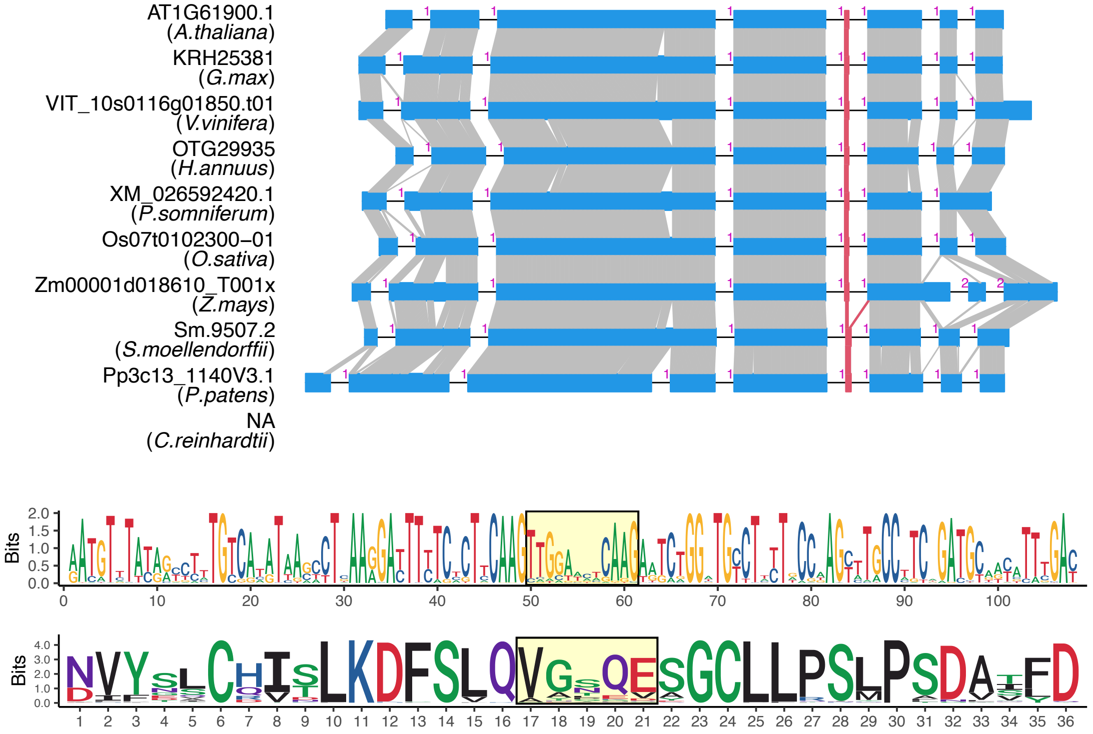

Supplementary Fig. 36: Multiple sequence alignments of microexon-containing genes and the homologs in 10 plant species, and DNA and AA sequence logos in microexon-tag Cluster 30.

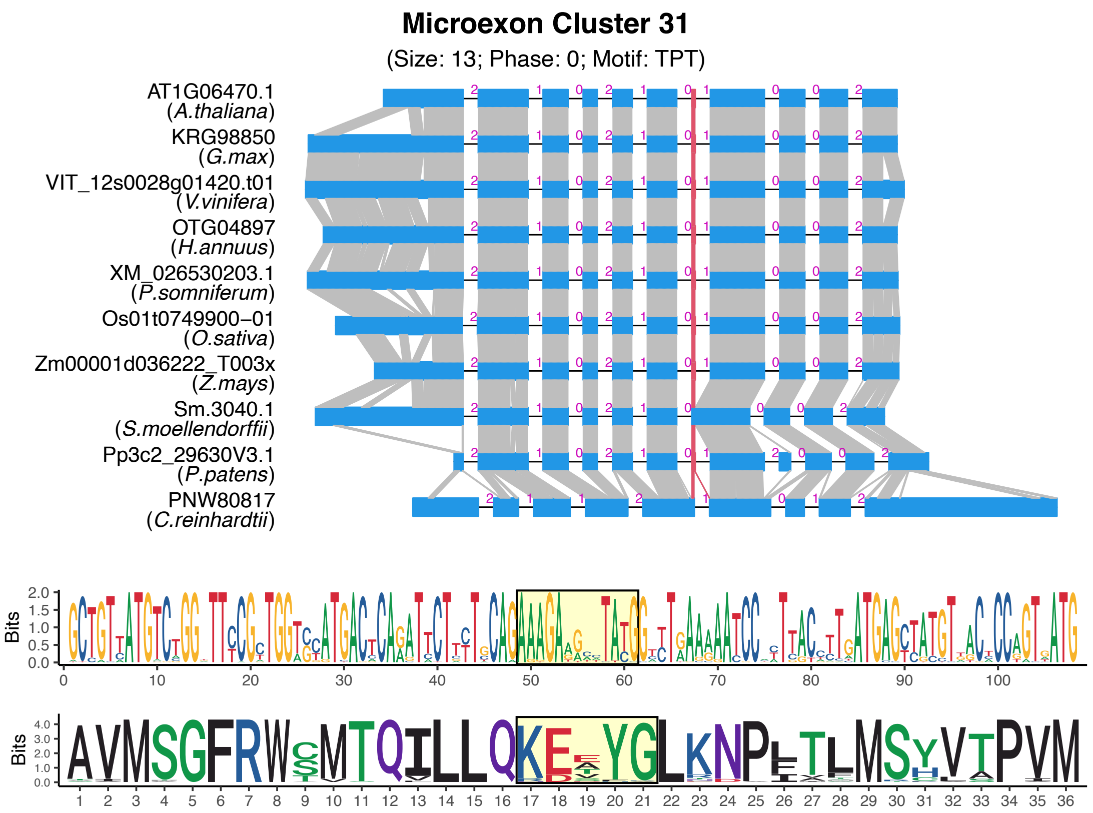

Supplementary Fig. 37: Multiple sequence alignments of microexon-containing genes and the homologs in 10 plant species, and DNA and AA sequence logos in microexon-tag Cluster 31.

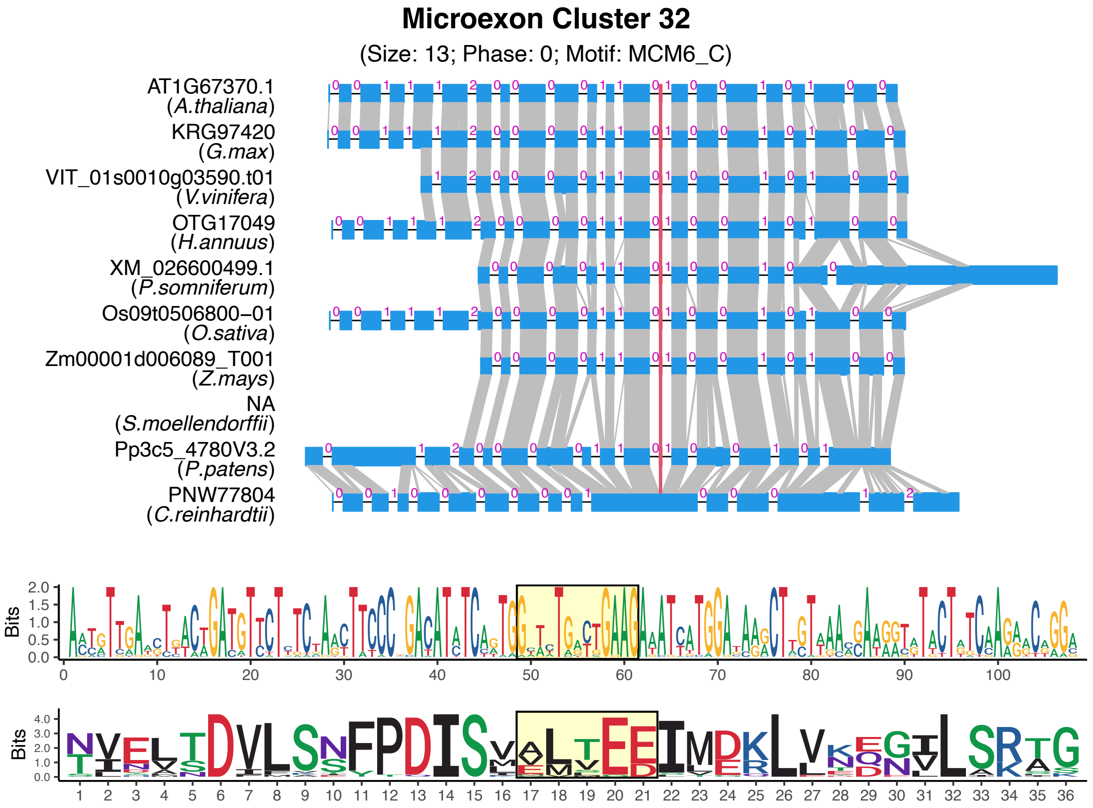

Supplementary Fig. 38: Multiple sequence alignments of microexon-containing genes and the homologs in 10 plant species, and DNA and AA sequence logos in microexon-tag Cluster 32.

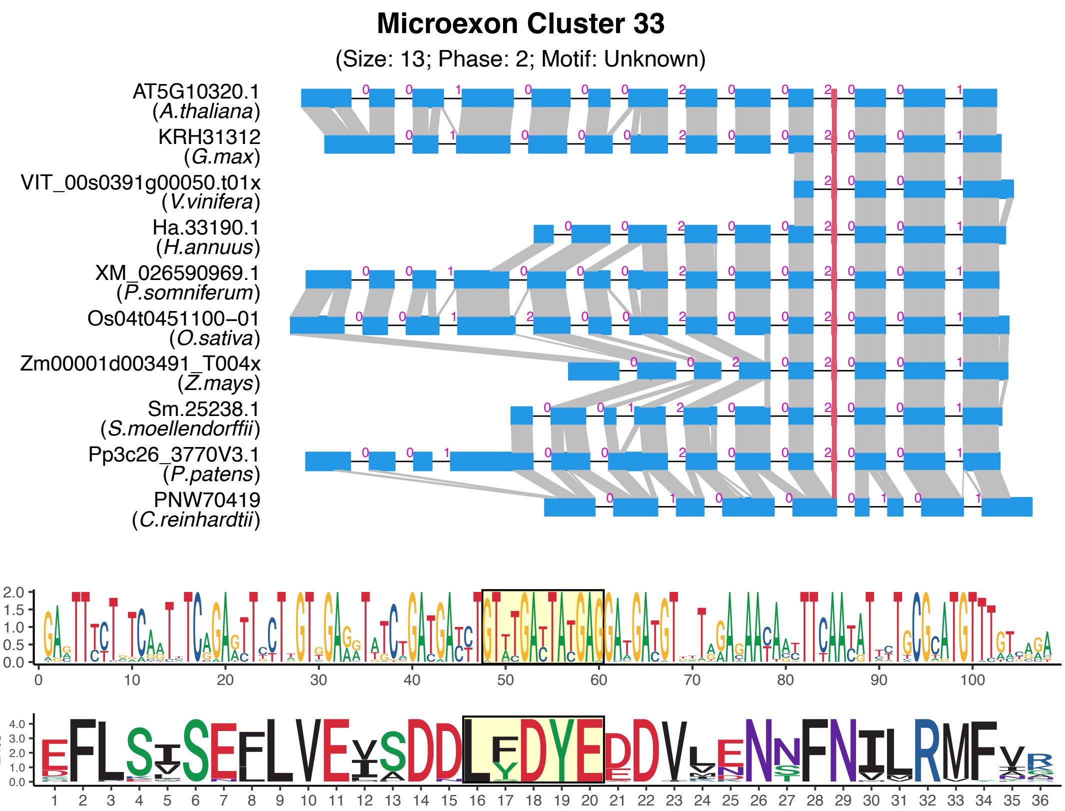

Supplementary Fig. 39: Multiple sequence alignments of microexon-containing genes and the homologs in 10 plant species, and DNA and AA sequence logos in microexon-tag Cluster 33.

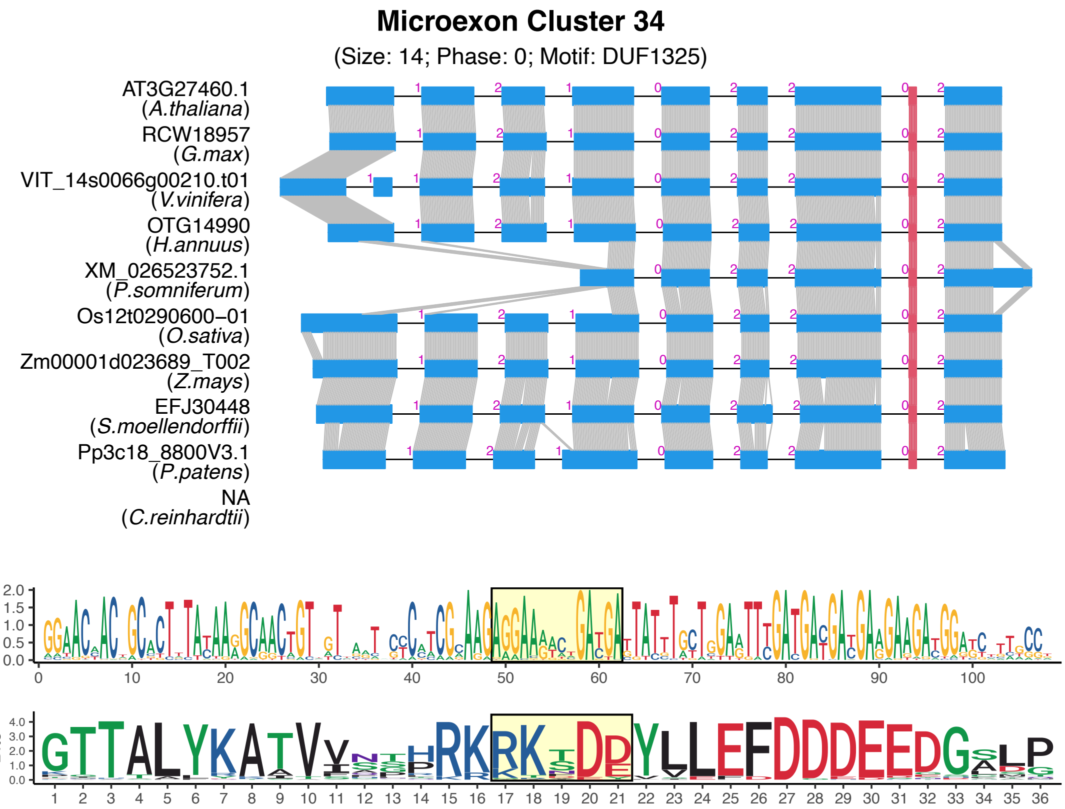

Supplementary Fig. 40: Multiple sequence alignments of microexon-containing genes and the homologs in 10 plant species, and DNA and AA sequence logos in microexon-tag Cluster 34.

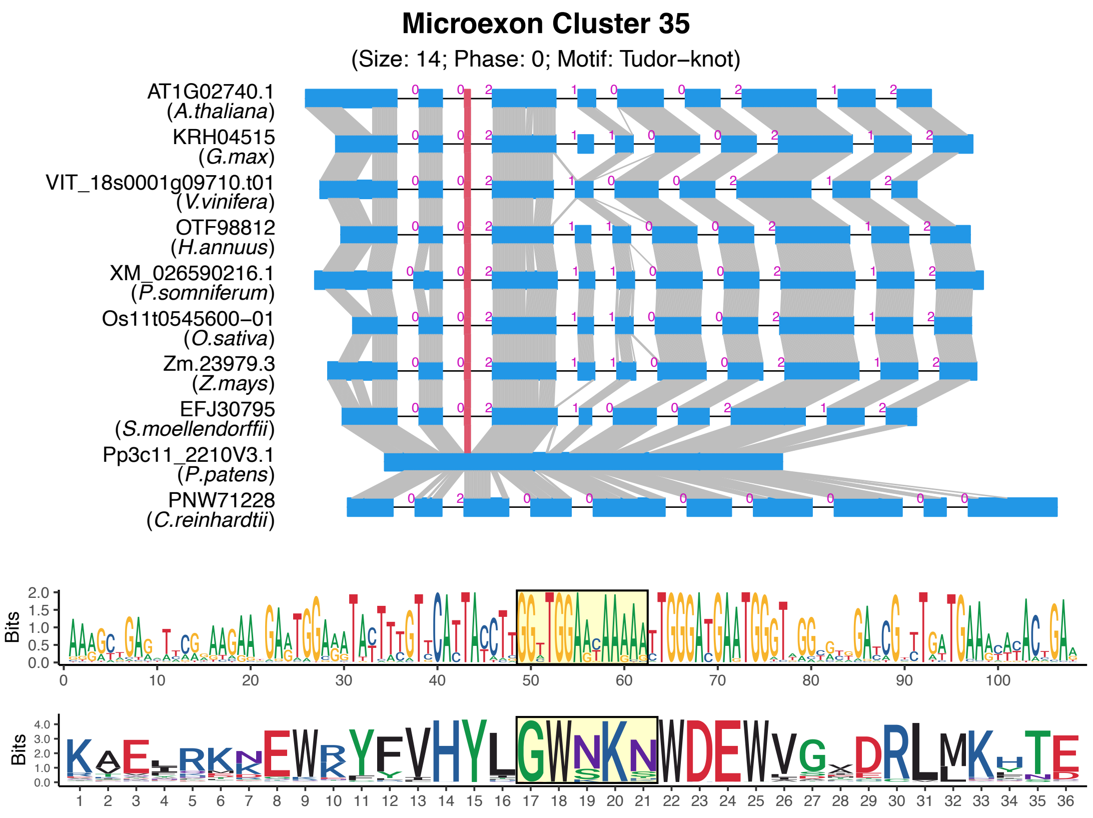

Supplementary Fig. 41: Multiple sequence alignments of microexon-containing genes and the homologs in 10 plant species, and DNA and AA sequence logos in microexon-tag Cluster 35.

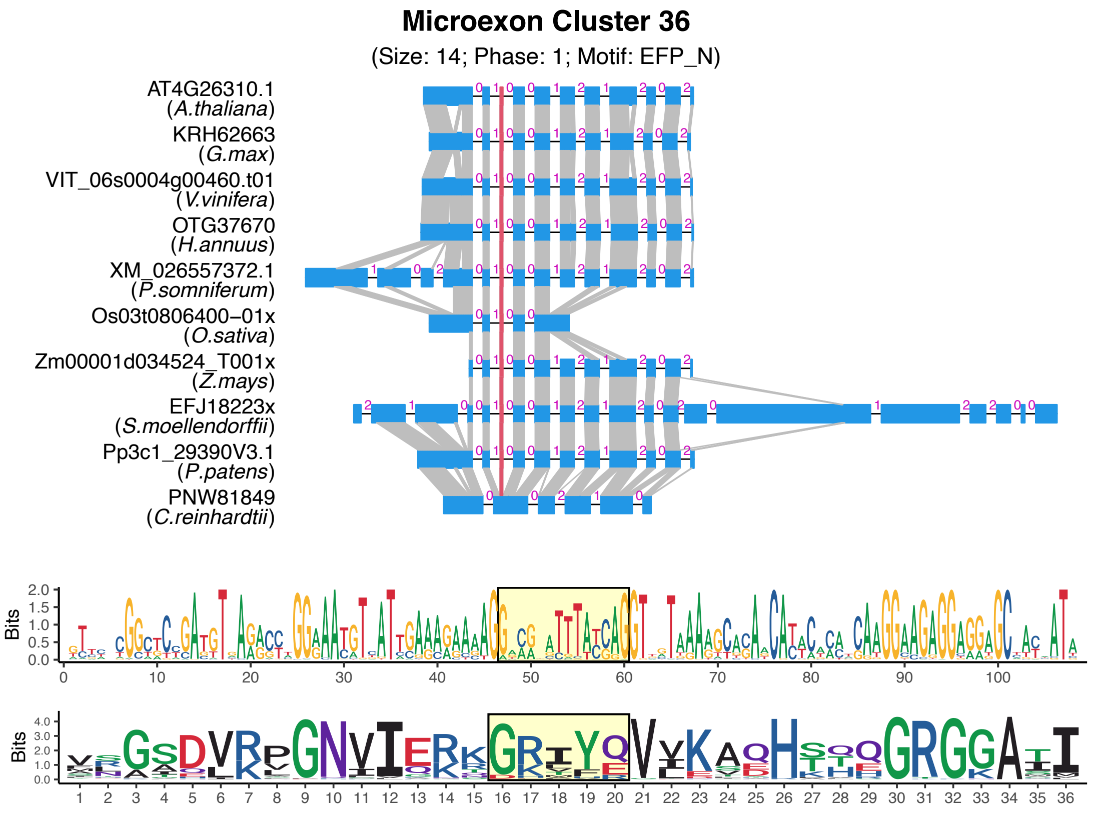

Supplementary Fig. 42: Multiple sequence alignments of microexon-containing genes and the homologs in 10 plant species, and DNA and AA sequence logos in microexon-tag Cluster 36.

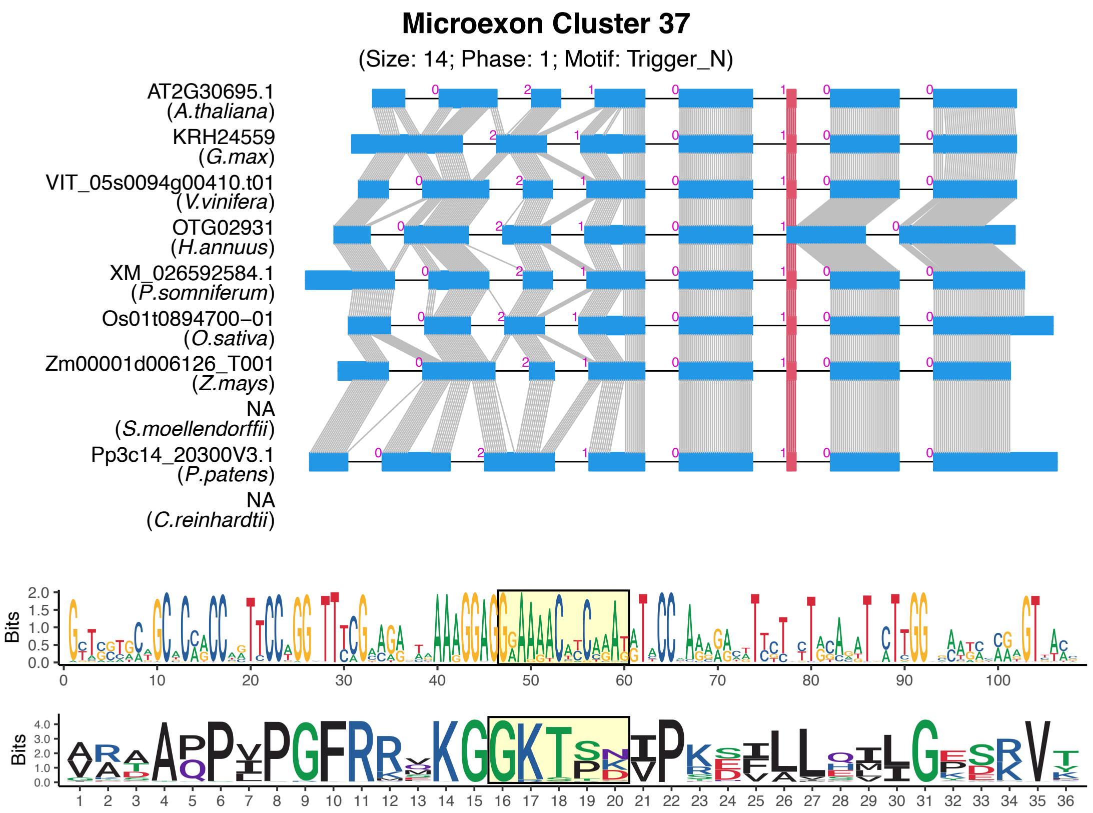

Supplementary Fig. 43: Multiple sequence alignments of microexon-containing genes and the homologs in 10 plant species, and DNA and AA sequence logos in microexon-tag Cluster 37.

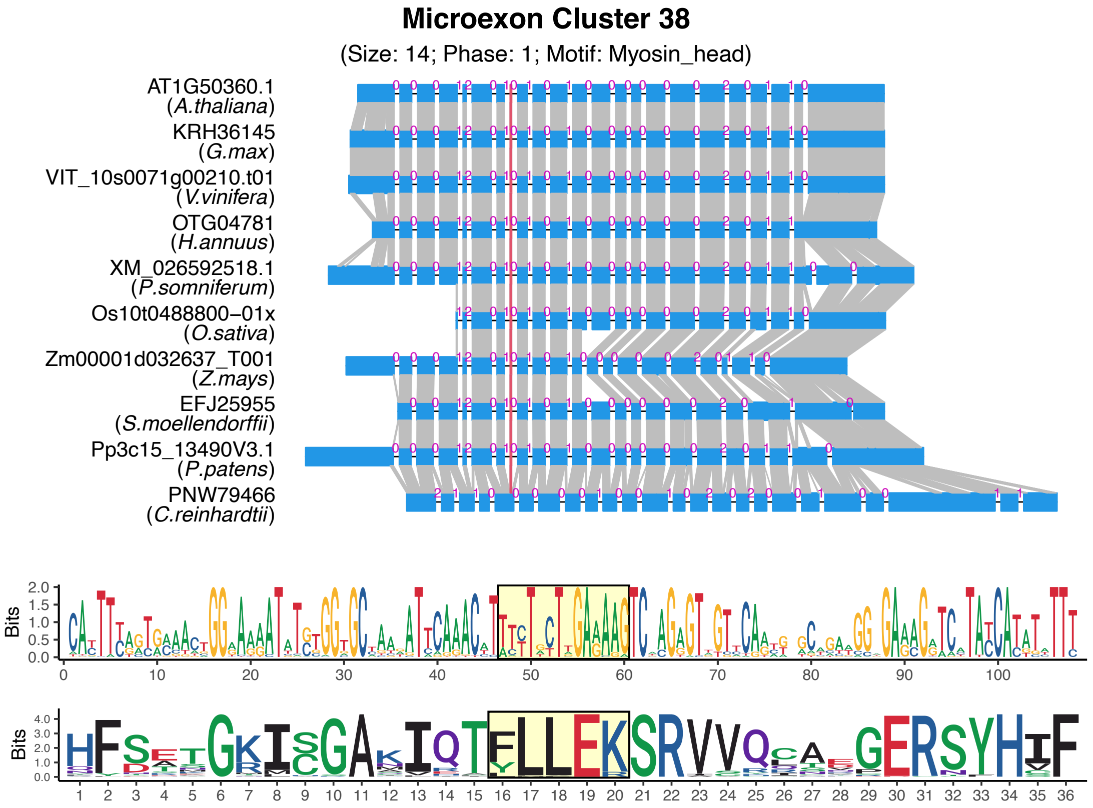

Supplementary Fig. 44: Multiple sequence alignments of microexon-containing genes and the homologs in 10 plant species, and DNA and AA sequence logos in microexon-tag Cluster 38.

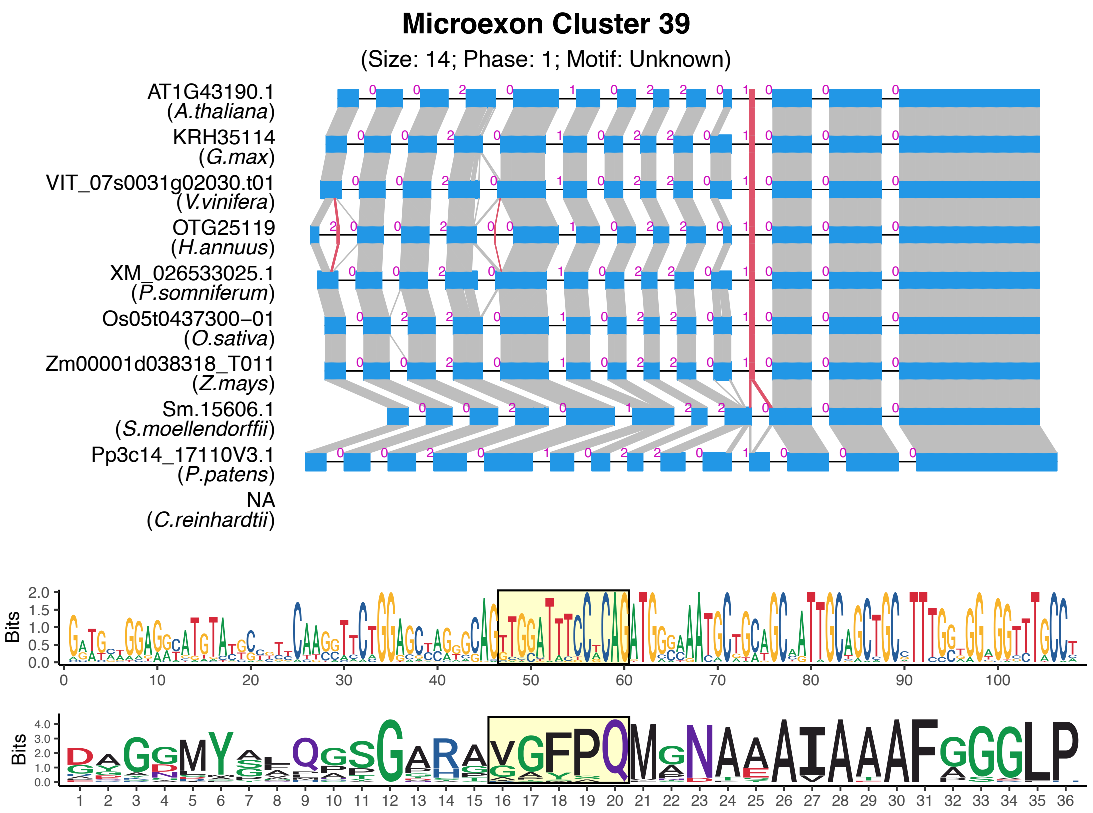

Supplementary Fig. 45: Multiple sequence alignments of microexon-containing genes and the homologs in 10 plant species, and DNA and AA sequence logos in microexon-tag Cluster 39.

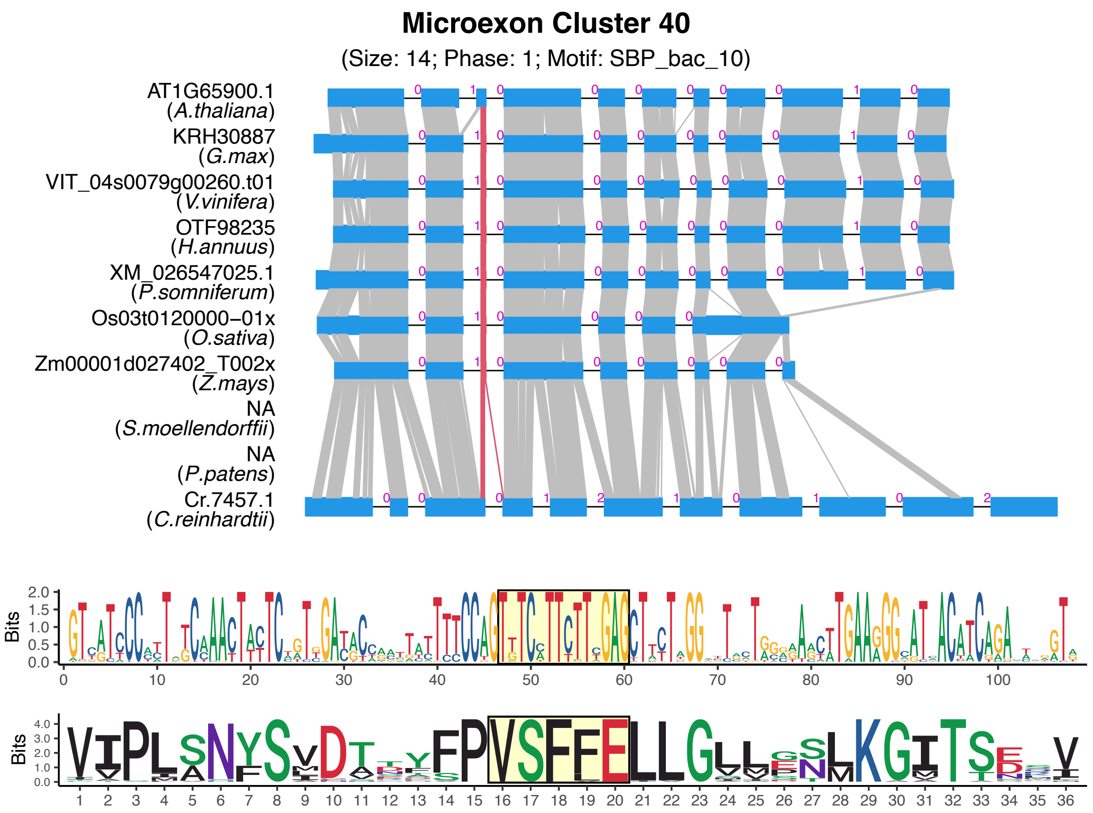

Supplementary Fig. 46: Multiple sequence alignments of microexon-containing genes and the homologs in 10 plant species, and DNA and AA sequence logos in microexon-tag Cluster 40.

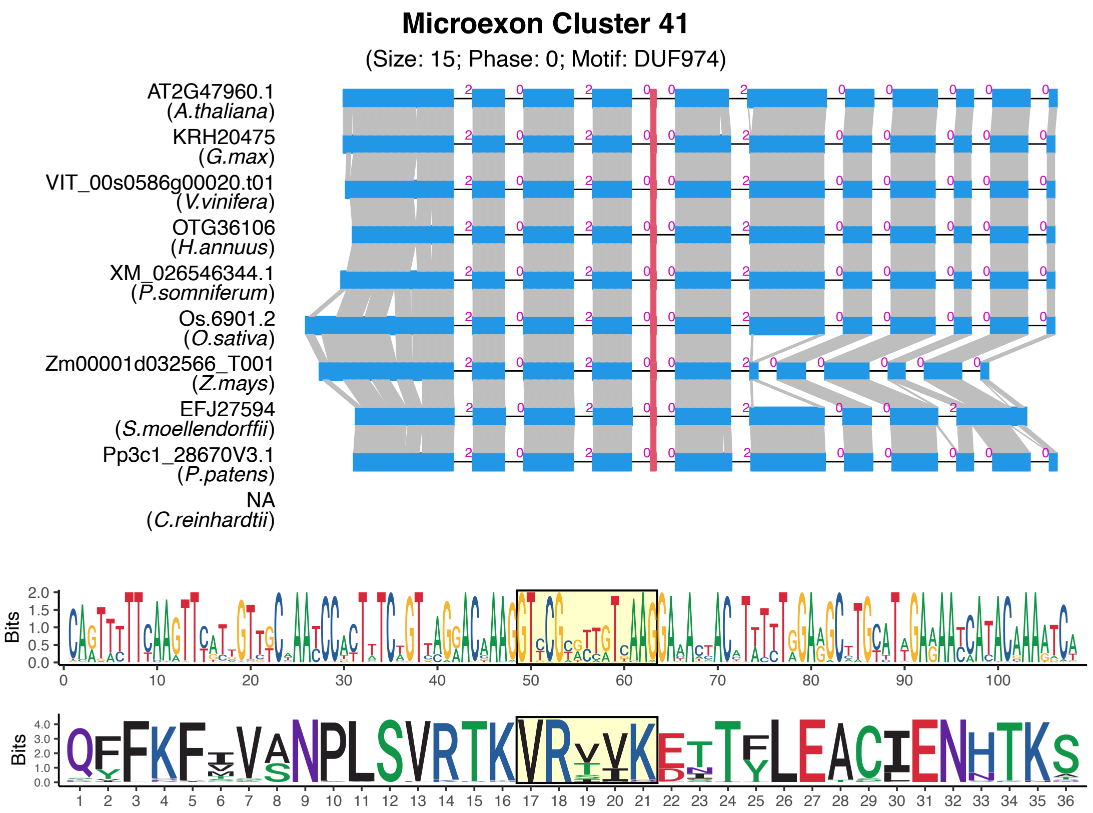

Supplementary Fig. 47: Multiple sequence alignments of microexon-containing genes and the homologs in 10 plant species, and DNA and AA sequence logos in microexon-tag Cluster 41.

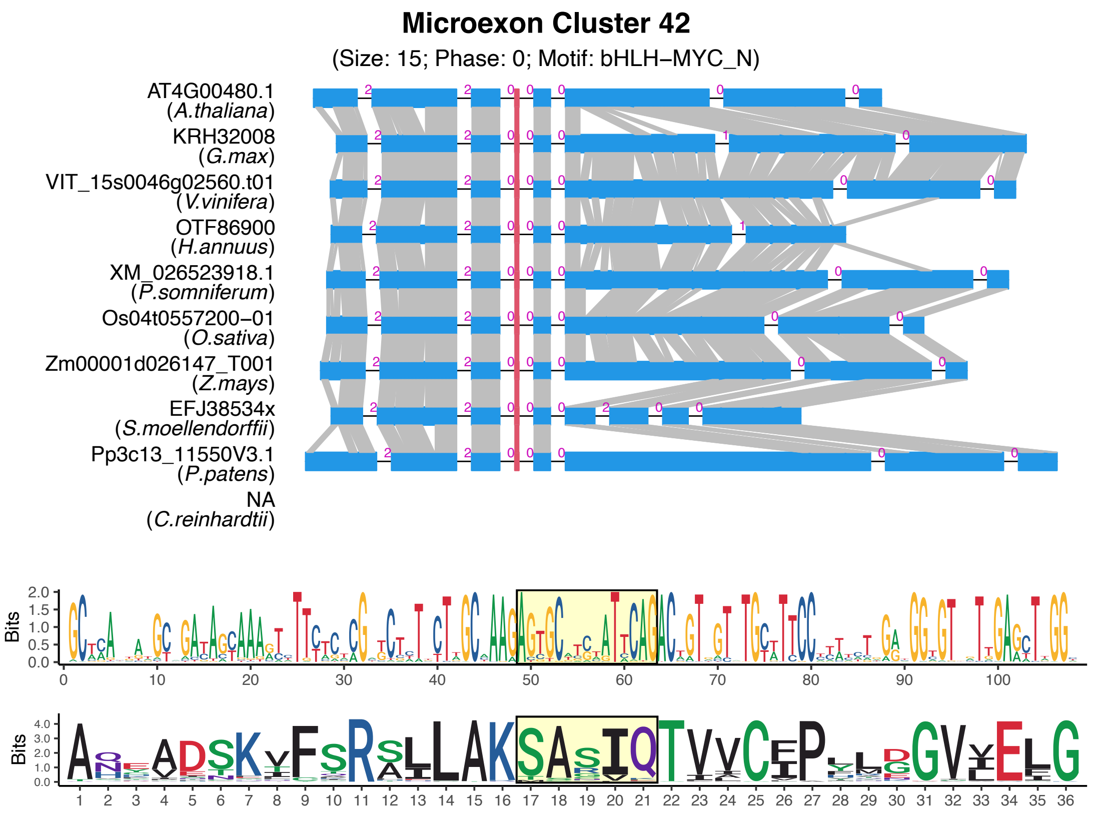

Supplementary Fig. 48: Multiple sequence alignments of microexon-containing genes and the homologs in 10 plant species, and DNA and AA sequence logos in microexon-tag Cluster 42.

Microexon Cluster 43

(Size: 15; Phase: 0; Motif: Unknown)

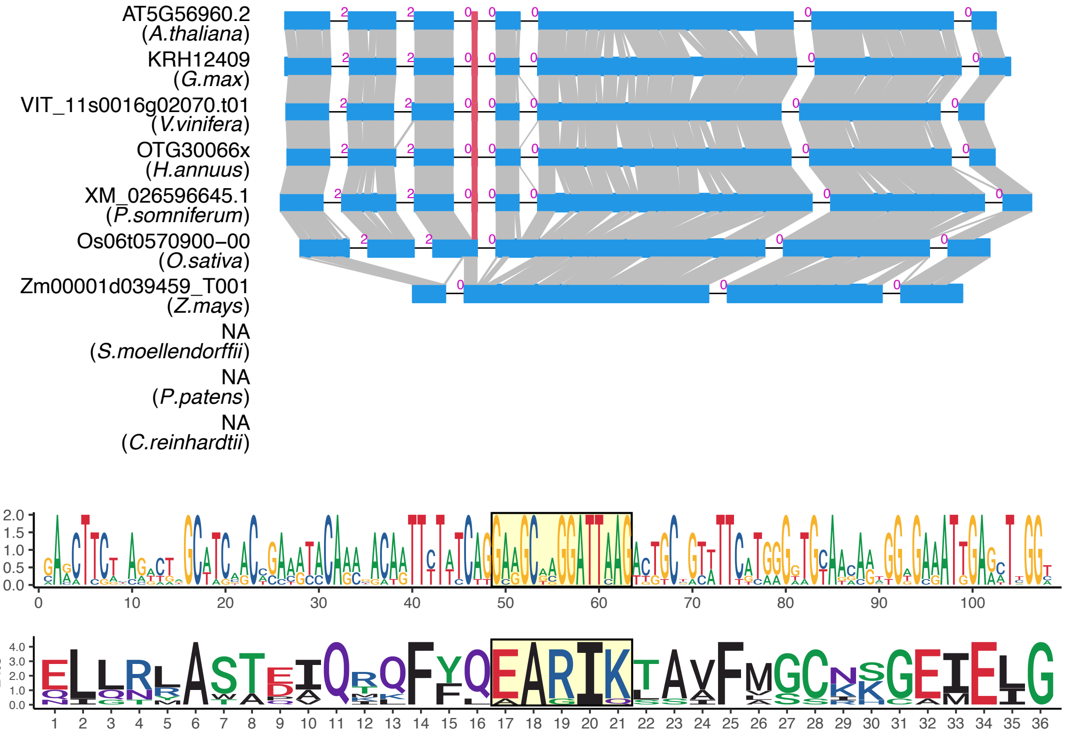

Supplementary Fig. 49: Multiple sequence alignments of microexon-containing genes and the homologs in 10 plant species, and DNA and AA sequence logos in microexon-tag Cluster 43.

Microexon Cluster 44

(Size: 15; Phase: 1; Motif: Unknown)

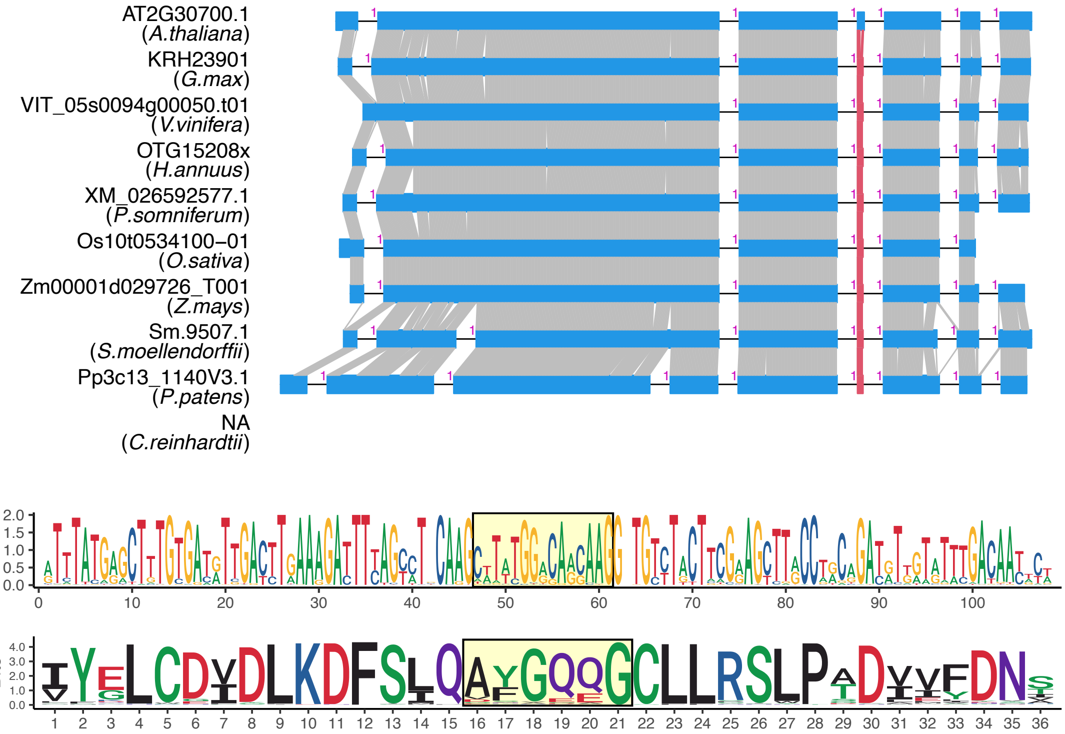

Supplementary Fig. 50: Multiple sequence alignments of microexon-containing genes and the homologs in 10 plant species, and DNA and AA sequence logos in microexon-tag Cluster 44.

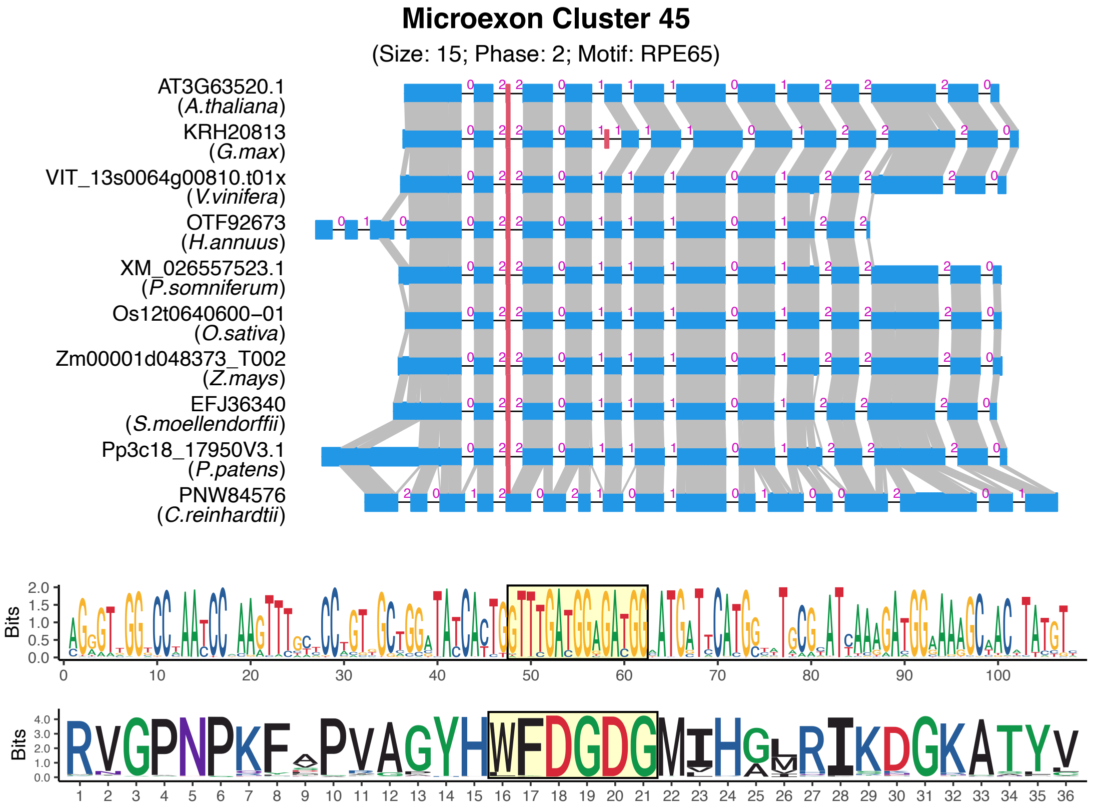

Supplementary Fig. 51: Multiple sequence alignments of microexon-containing genes and the homologs in 10 plant species, and DNA and AA sequence logos in microexon-tag Cluster 45.

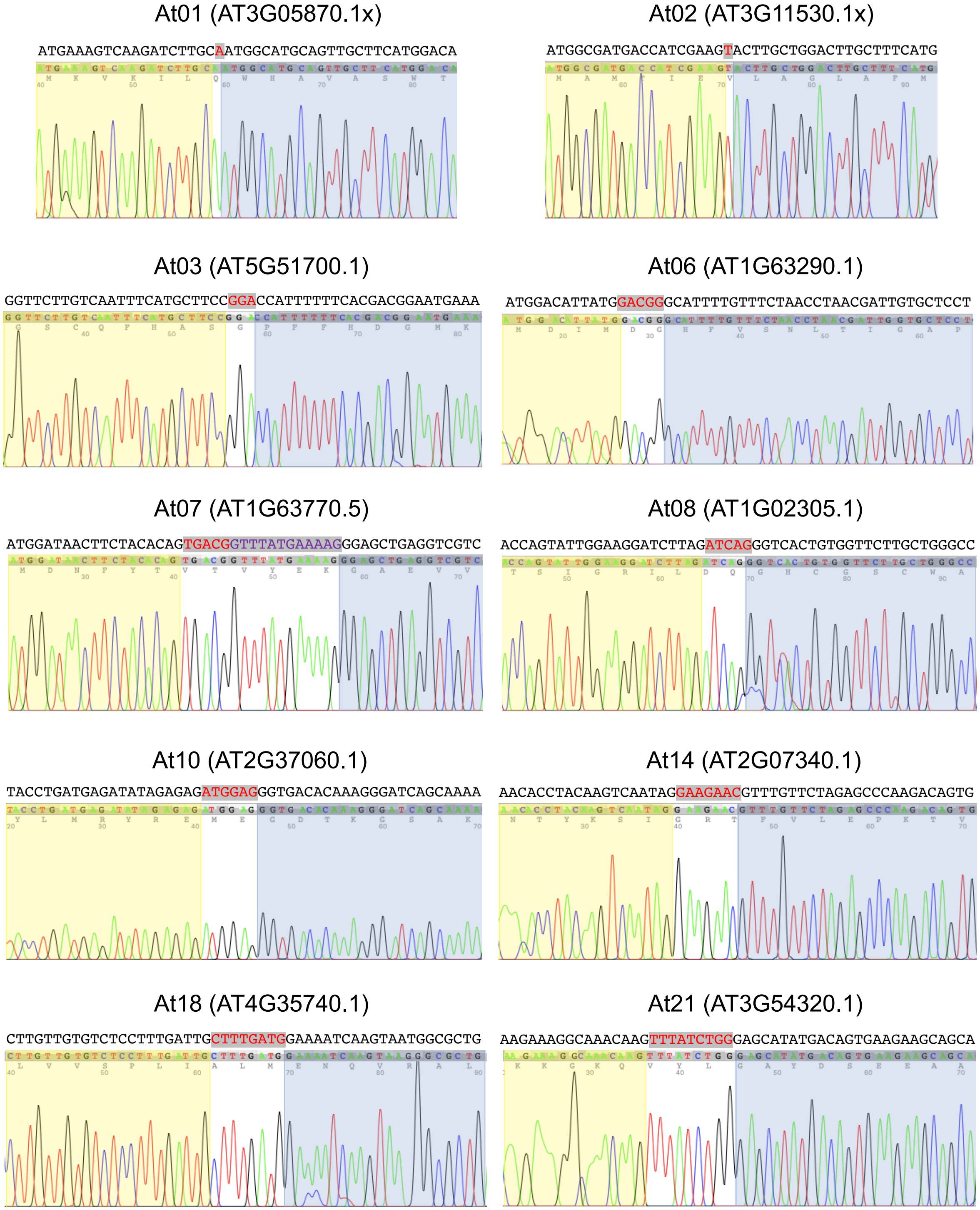

Supplementary Fig. 52: Microexon validation by RT-PCR sequencing in Arabidopsis.

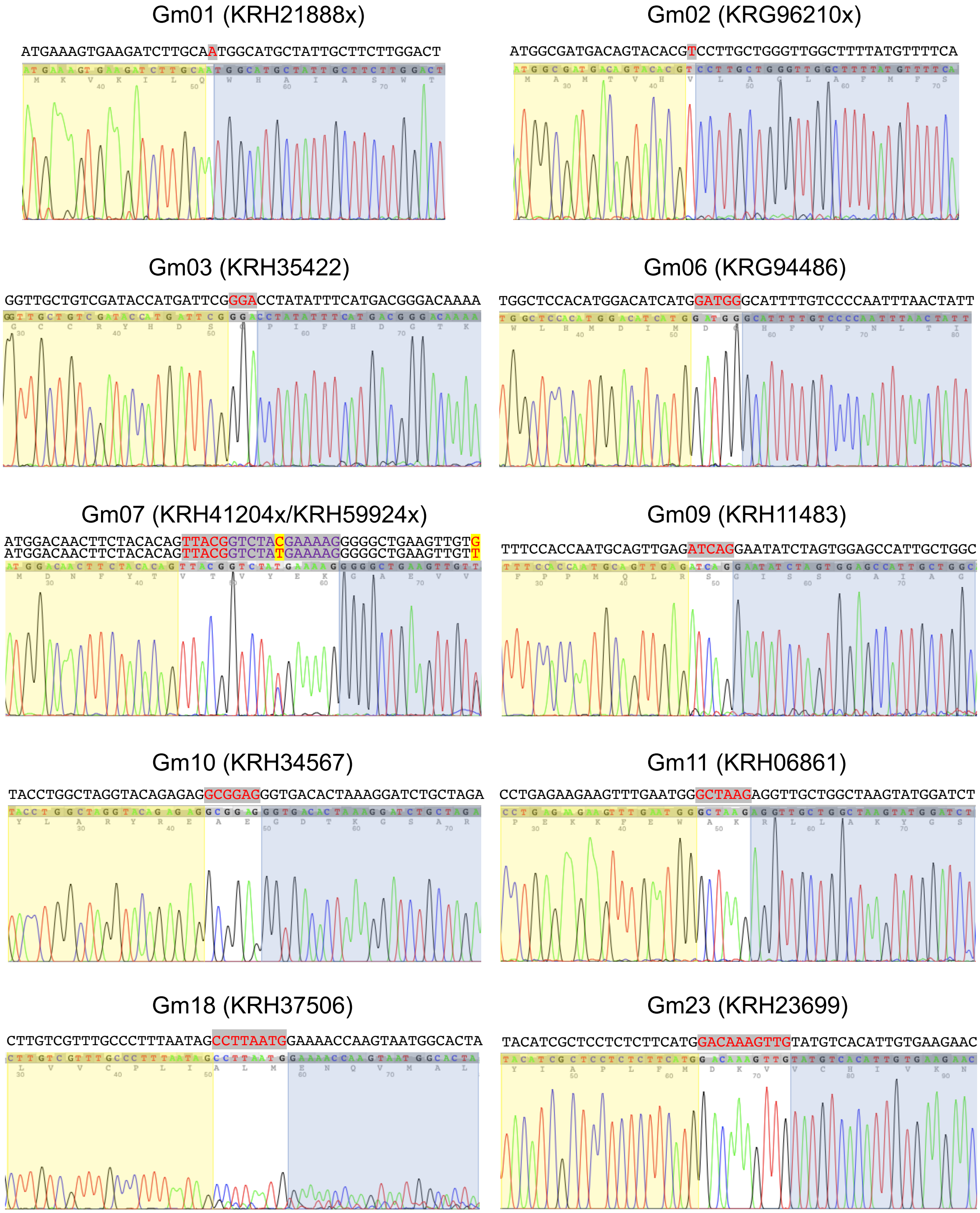

Supplementary Fig. 53: Microexon validation by RT-PCR sequencing in soybean.

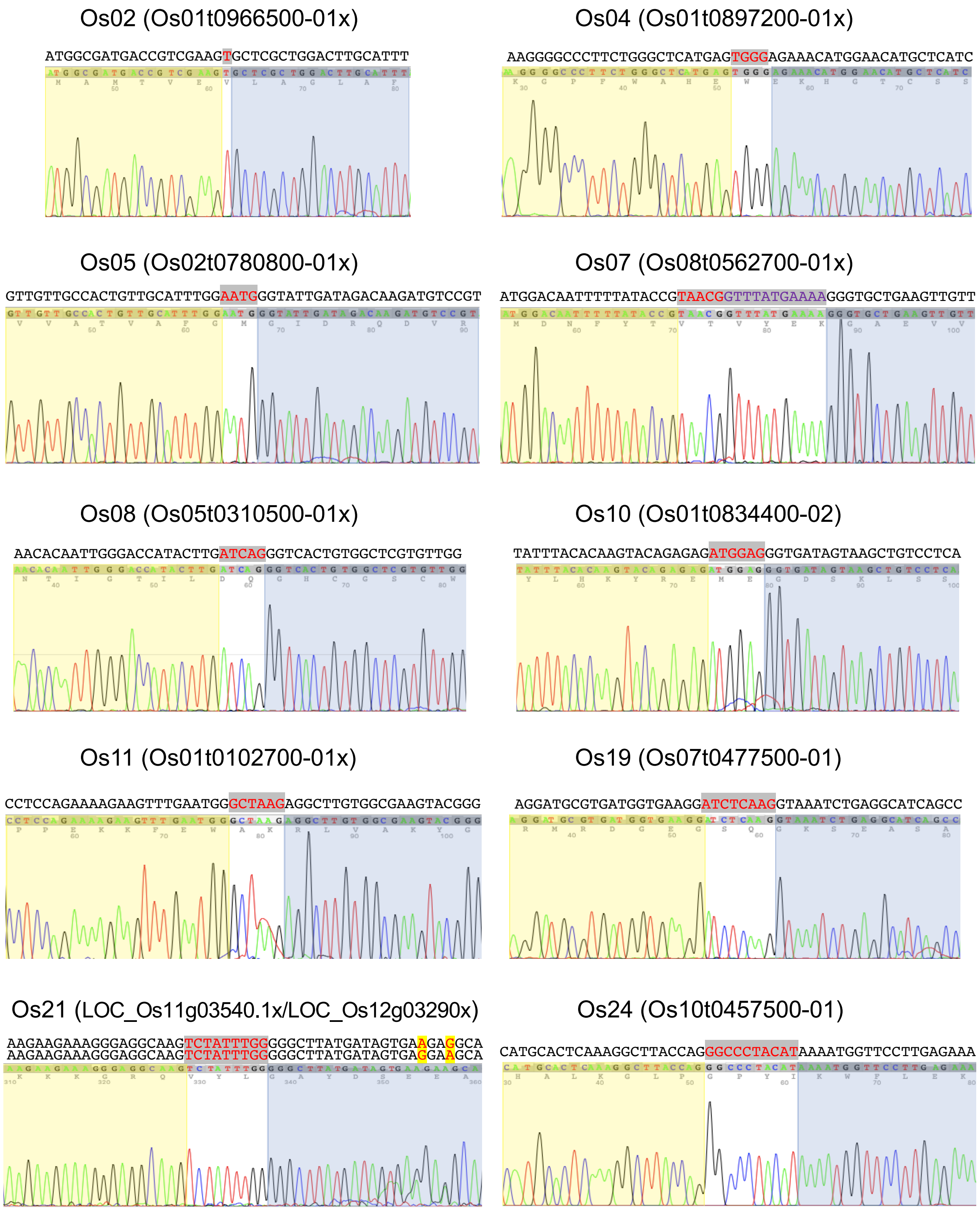

Supplementary Fig. 54: Microexon validation by RT-PCR sequencing in rice.

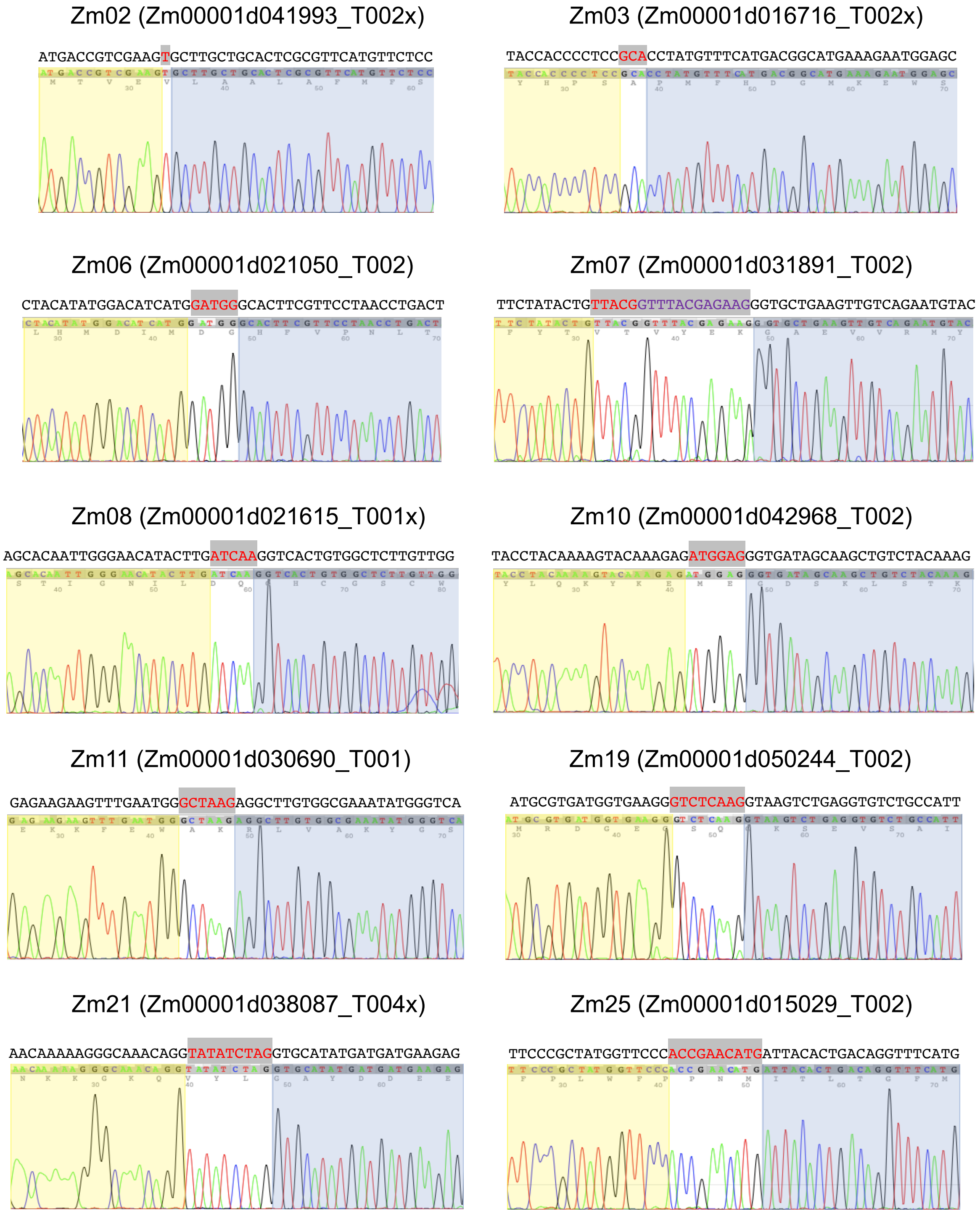

Supplementary Fig. 55: Microexon validation by RT-PCR sequencing in maize.

a

```
                                      TM1                                   TM2
VPS55_YEAST    MME-----FKVSPLTKIISLSGFLALGFLLVILSCALFHNYYPLFDILIFLLAPIPNTIF 55
AT1G32410.1    MADVPGYLRTCLDMGKIAFLAILVSTGIVLQILACALFNNWWPMLSVIMYVLLPMPLLFF 60
AT3G11530.1    ----------------------MFSSSILLQILACAIYGNWWPMLSALMYVVVPMPCMFF 38
AT3G11530.1x   ---------MAMTIEVLAGLAFMFSSSILLQILACAIYGNWWPMLSALMYVVVPMPCMFF 51
                                      :.:  .::* **:**:: *::*::. ::::: *:*  :*
               1-nt microexon
                  encoding                  TM3                             TM4
VPS55_YEAST    NAGNKYHTSDFMSDSSNTGQDLAHFLTGMLVTSGIALPVVFYHCQLIGHLSCIMCMIGGL 115
AT1G32410.1    GGSDS---TSLFNESDNSWINAAKFLTGASAVGSVAIPSILKHAGLIGWGALALDLSSYV 117
AT3G11530.1    GGGST---QFLISRDGGGWIDAAKFLTGASTVGSLAIPIILRHAQMIETGAMLIEFTSFF 95
AT3G11530.1x   GGGST---QFLISRDGGGWIDAAKFLTGASTVGSLAIPIILRHAQMIETGAMLIEFTSFF 108
               .....     ::. ...   : *:****  ....:*:* :: *. :*   :  : : . .

VPS55_YEAST    IIYSSIVIFKWFFKKDFNEDDSLFG-- 140
AT1G32410.1    VFLVAILGY----ICIGDASDNYYSYI 140
AT3G11530.1    IFICTVMCF----HRASL-DDDW---- 113
AT3G11530.1x   IFICTVMCF----HRASL-DDDW---- 126
               ::  ::: :           .*.
```

b

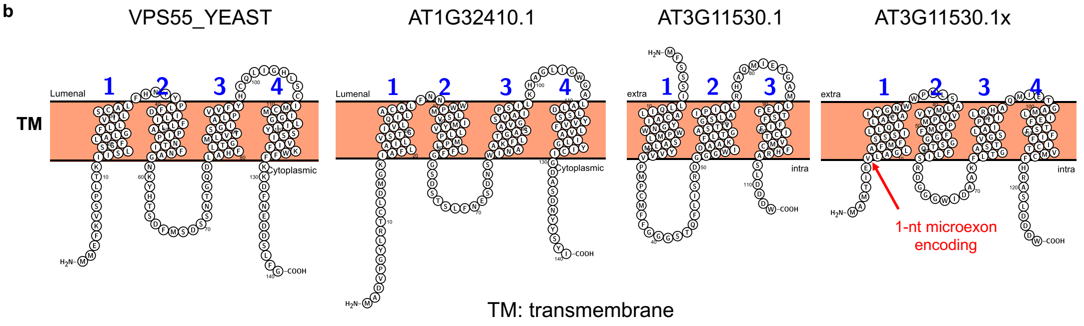

Supplementary Fig. 56: VPS55 protein transmembrane prediction. a, Multiple sequence alignment of VPS55 proteins in *Saccharomyces cerevisiae* (VPS55_YEAST), full-length VPS55-1 by considering the discovered 1-nt microexon (AT3G11530.1x), annotated VPS55-1 (AT3G11530.1) and another VPS55 without microexons VPS55-2 (AT1G32410.1) in *Arabidopsis*. The transmembrane regions are highlighted in purple. **b**, Transmembrane helix prediction from Protter (http://wlab.ethz.ch/protter/start/). AT3G11530.1 has only three transmembrane helices and its N-term is located in the extracellular side, while the other full-length proteins, (VPS55_YEAST, AT1G32410.1, and AT3G11530.1x), all have four transmembrane helices and the N-term of the protein is in intracellular side (cytoplasm).

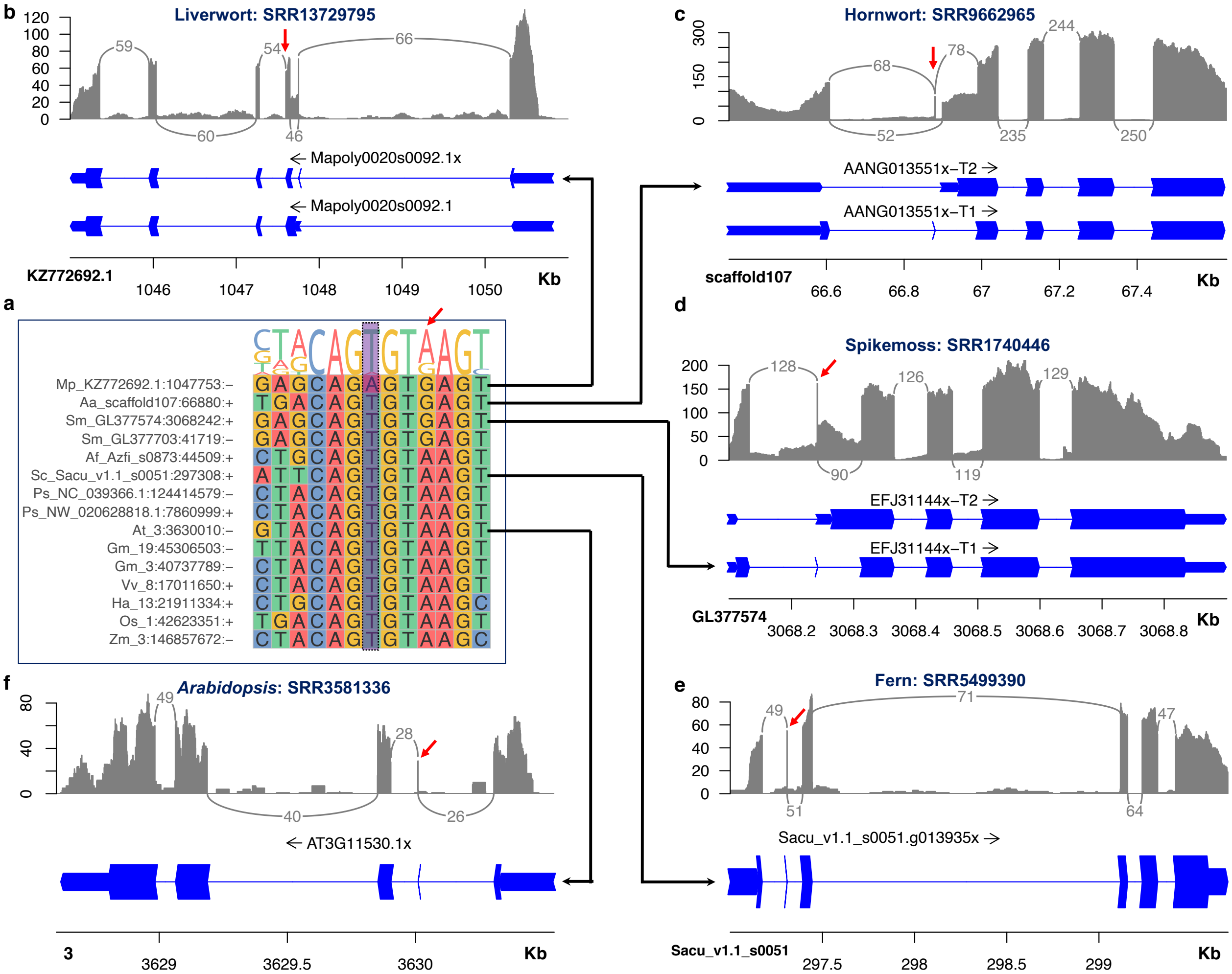

Supplementary Fig. 57: Comparison of the splicing around the 1-nt microexon in VPS55. a, Comparison of splice site sequences flanking the 1-nt microexons in *VPS55*. The highlighted purple box indicates the 1-nt microexon and the red arrow indicates the position of the splice donor site, changed from “G” to “A”, which causes the 1-nt microexon to gain a stronger splice site. **b**, RNA-seq read depth and two assembled transcripts for the single copy *VPS55* in *Marchanta polymorpha* (liverwort): one has the microexon and the other lost the microexon due to intron retention. **c**, RNA-seq read depth and two assembled transcripts for the single copy *VPS55* in the *Anthoceros angustus* (hornwort). **d**, RNA-seq read depth and two assembled transcripts for one copy of *VPS55* in *Selaginella*. **e**, RNA-seq read depth and one assembled transcript in *Salvinia cucullata* (fern). **f**, RNA-seq read depth and one assembled transcript in *Arabidopsis*. The flanking introns of the 1-nt microexon were nearly fully spliced in ferns and *Arabidopsis*. In **b-f**, arcs indicate junctions and the numbers on them indicate junction counts. Red arrows indicate the 1-nt microexon.

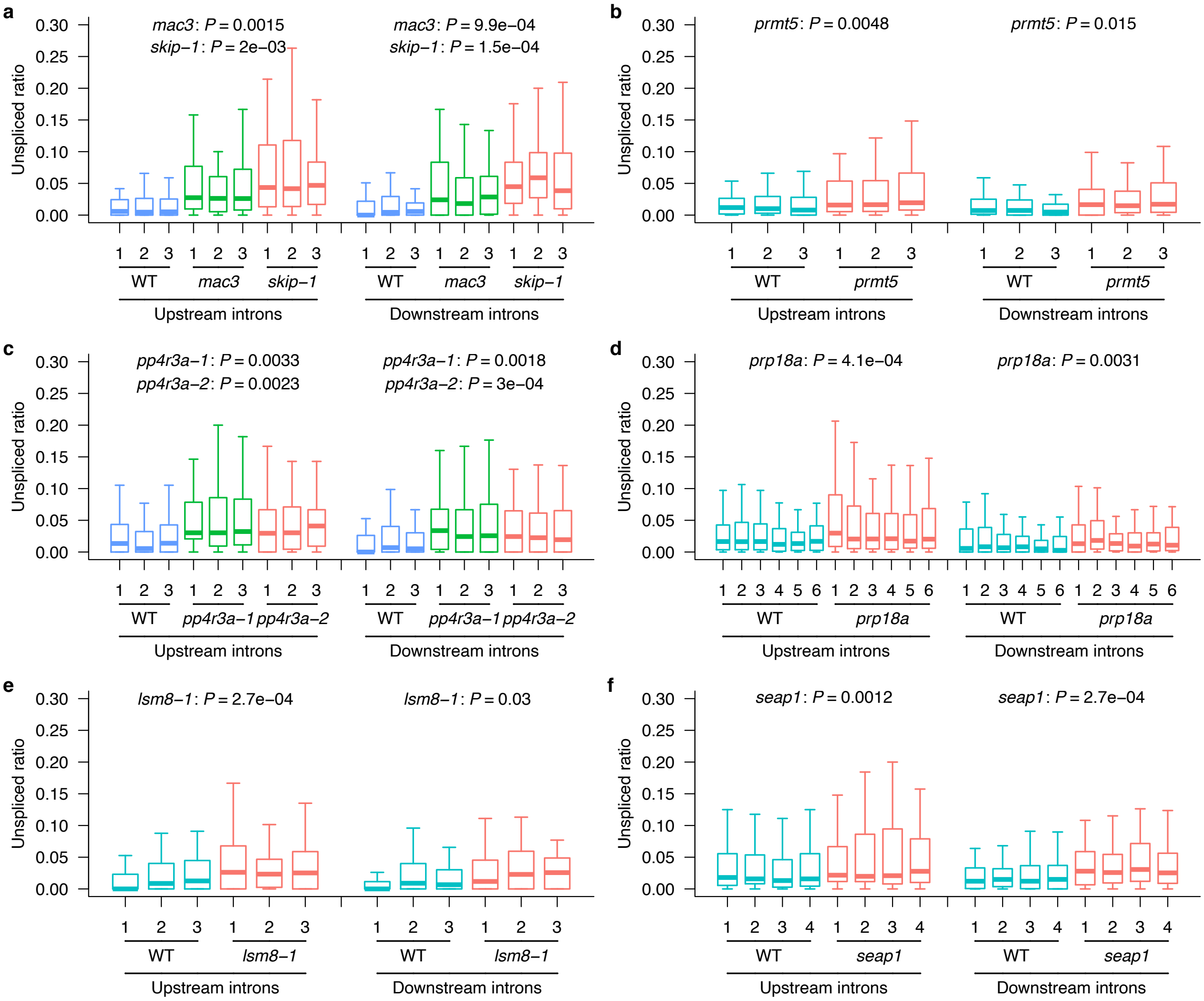

Supplementary Fig. 58: Distribution of the unspliced ratio of introns surrounding microexons in different mutants. **a**, *mac3* and *skip* (NCBI project: PRJNA374393). **b**, *prmt5* (NCBI project: PRJEB15204). **c**, *pp4r3a-1* and *pp4r3a-2* (NCBI project: PRJNA497374). **d**, *prp18a* (NCBI project: PRJNA412787). **e**, *lsm8-1* (NCBI project: PRJNA513852). **f**, *seap1* (NCBI project: PRJNA721214). For the box plots, the bounds of a box show the interquartile range (IQR), the center line in the box shows the median, and the whiskers extend to no further than 1.5 × IQR from the box bounds. The intron unspliced ratio was estimated based on the percentage of intron retention (PIR) value according to Jia et al.[25]. The *P*-values are the results of a one-sided t-test on that the unspliced ratios in the mutants are greater than those in wild types (WT). 1, 2, 3 *etc*. indicate replicates. Upstream introns and downstream introns are 5' and 3' flanking introns adjacent to the microexons, respectively. Only constitutively spliced introns with the unspliced ratio ≤ 0.2 in wild type samples were used in the analysis.

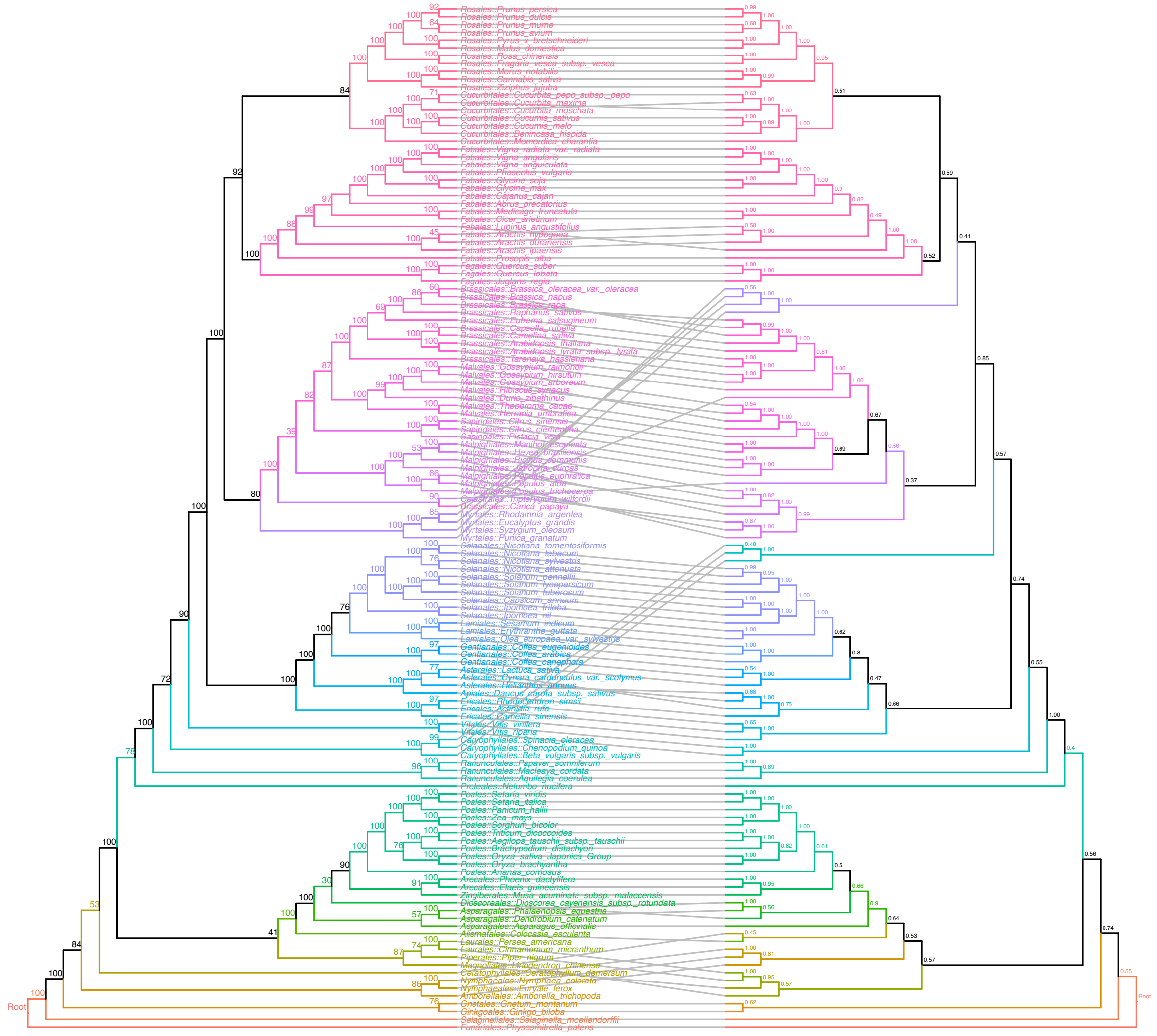

Supplementary Fig. 59: Comparison of phylogenetic trees constructed by two different methods. Left, Method 1; Right, Method 2 (See Methods for details).
