# Supplementary material for: Pervasive misannotation of microexons that are evolutionarily conserved and crucial for gene function in plants
Source: Nat Commun. 2022 Feb 10;13:820. doi: 10.1038/s41467-022-28449-8 (PMC8831610; doi:10.1038/s41467-022-28449-8)
Supplement: Supplementary file 3 — Description of Additional Supplementary Files [file 41467_2022_28449_MOESM3_ESM.pdf]

Description of Additional Supplementary Files

File Name: Supplementary Data 1
Description: SRA accession numbers of RNA-seq samples in 10 plant species.

File Name: Supplementary Data 2
Description: RT-PCR and sequencing primers used for microexon validation in four plant species.
