# Supplementary material for: Pervasive misannotation of microexons that are evolutionarily conserved and crucial for gene function in plants
Source: Nat Commun. 2022 Feb 10;13:820. doi: 10.1038/s41467-022-28449-8 (PMC8831610; doi:10.1038/s41467-022-28449-8)
Supplement: Supplementary file 6 — Reporting Summary [file 41467_2022_28449_MOESM6_ESM.pdf]

nature portfolio

Corresponding author(s): Chi Zhang, Jeffrey Mower, Bin Yu, Weibo Xie

Last updated by author(s): Jan 20, 2021

Reporting Summary

Nature Portfolio wishes to improve the reproducibility of the work that we publish. This form provides structure for consistency and transparency in reporting. For further information on Nature Portfolio policies, see our Editorial Policies and the Editorial Policy Checklist.

Statistics

For all statistical analyses, confirm that the following items are present in the figure legend, table legend, main text, or Methods section.

n/a	Confirmed	
☐	☒	The exact sample size (*n*) for each experimental group/condition, given as a discrete number and unit of measurement
☐	☒	A statement on whether measurements were taken from distinct samples or whether the same sample was measured repeatedly
☐	☒	The statistical test(s) used AND whether they are one- or two-sided<br>*Only common tests should be described solely by name; describe more complex techniques in the Methods section.*
☒	☐	A description of all covariates tested
☒	☐	A description of any assumptions or corrections, such as tests of normality and adjustment for multiple comparisons
☐	☒	A full description of the statistical parameters including central tendency (e.g. means) or other basic estimates (e.g. regression coefficient) AND variation (e.g. standard deviation) or associated estimates of uncertainty (e.g. confidence intervals)
☐	☒	For null hypothesis testing, the test statistic (e.g. *F*, *t*, *r*) with confidence intervals, effect sizes, degrees of freedom and *P* value noted<br>*Give P values as exact values whenever suitable.*
☒	☐	For Bayesian analysis, information on the choice of priors and Markov chain Monte Carlo settings
☐	☒	For hierarchical and complex designs, identification of the appropriate level for tests and full reporting of outcomes
☒	☐	Estimates of effect sizes (e.g. Cohen's *d*, Pearson's *r*), indicating how they were calculated

Our web collection on statistics for biologists contains articles on many of the points above.

Software and code

Policy information about availability of computer code

Data collection	A pipeline integrating STAR and OLego was developed and optimized to identity microexons from short read RNA-seq data. STAR (version 2.7.1a), HISAT2 (version 2.1.0), and OLego (version 1.1.5) were used for comparison of different methods for microexon detection. All codes for microexon discovery, microexon clustering, and microexon modeling are deposited in Zenodo (DOI:10.5281/zenodo.5815987) and also available at GitHub (https://github.com/yuhuihui2011/MEPsuite). MEPmodeler, an R package for microexon modeling in plant genomes, is deposited in Zenodo (DOI:10.5281/zenodo.5816080) and also available at GitHub (https://github.com/yuhuihui2011/MEPmodeler).

Data analysis	R package Polyester (version 1.26.0) was used to simulate RNA-seq reads.<br><br>MEME was used to conduct differential enrichment mode analysis (http://meme-suite.org/tools/meme).<br><br>BLASTN (version 2.10.1) was used to search NCBI plant EST database.<br><br>The function getSeq from the R package BSgenome (version 1.58.0) was used to extract transcript sequences, The R package ORFik (version 1.10.7) was used to get ORF locations on the reference genomes with the function findMapORFs. The function translate from the Biostrings package (version 2.58.0) was used to translate CDS sequences into protein sequences. The online tool GenomeNet was used to find protein motifs with default parameters (https://www.genome.jp/tools/motif/). The R package ggseqlogo (version 0.1) and ggplot2 (version 3.3.3) were used for visualization.<br><br>Phylogenetic tree construction based on microexon prediction: Method 1, The phylogenetic tree was constructed based on the concatenated large DNA strings of all species using IQ-TREE2 (version 2.1.2) with 1000 ultrafast bootstrap replicates; Method 2, The phylogenetic tree in each cluster was generated by using IQ-TREE2 with 1000 ultrafast bootstrap replicates. The species tree was constructed based on all cluster trees using ASTRAL-Pro (version 1.1.5). The phylogenetic trees were manipulated and visualized using R package ape (version 5.4.1) and ggtree (version 2.4.1).

For manuscripts utilizing custom algorithms or software that are central to the research but not yet described in published literature, software must be made available to editors and reviewers. We strongly encourage code deposition in a community repository (e.g. GitHub). See the Nature Portfolio guidelines for submitting code & software for further information.

Data

Policy information about availability of data

All manuscripts must include a data availability statement. This statement should provide the following information, where applicable:

- Accession codes, unique identifiers, or web links for publicly available datasets
- A description of any restrictions on data availability
- For clinical datasets or third party data, please ensure that the statement adheres to our policy

A total of 990 RNA-seq datasets were collected from NCBI SRA for all 10 plants (https://www.ncbi.nlm.nih.gov/sra), and the accession numbers are available in Supplementary Data 1.

For the performance comparison of different mapping tools in microexon identification, two 50-bp and two 100-bp Illumina RNA-seq datasets were collected from Arabidopsis (SRA accessions: SRR3581695{https://www.ncbi.nlm.nih.gov/sra/?term=SRR3581695}, SRR3581709{https://www.ncbi.nlm.nih.gov/sra/?term=SRR3581709}, SRR14209167{https://www.ncbi.nlm.nih.gov/sra/?term=SRR14209167} and SRR14209168{https://www.ncbi.nlm.nih.gov/sra/?term=SRR14209168}) and rice (DRX000664{https://www.ncbi.nlm.nih.gov/sra/?term=DRX000664}, DRX000672{https://www.ncbi.nlm.nih.gov/sra/?term=DRX000672}, SRR5126147{https://www.ncbi.nlm.nih.gov/sra/?term=SRR5126147}, and SRR5126148{https://www.ncbi.nlm.nih.gov/sra/?term=SRR5126148}). RNA-seq datasets for microexon prediction validation included three 101-bp replicates (SRR8434771{https://www.ncbi.nlm.nih.gov/sra/?term=SRR8434771}, SRR8434772{https://www.ncbi.nlm.nih.gov/sra/?term=SRR8434772}, and SRR8434773{https://www.ncbi.nlm.nih.gov/sra/?term=SRR8434773}) from 4-day old roots of tomato (Solanum lycopersicum). RNA-seq datasets for post-transcriptional splicing analysis were from NCBI project accession PRJNA591665 {https://www.ncbi.nlm.nih.gov/sra/?term=PRJNA591665} (only Illumine RNA-seq used).

For phylogenetic tree construction, plant genome sequences were downloaded from NCBI plant RefSeq genome database (https://www.ncbi.nlm.nih.gov/refseq/, March 2021).

All the RNA-seq data, genome annotations, and genome sequences were obtained from public databases and their accession numbers are listed in Methods and supplementary files. Other data can be found in supplementary data and source data.

Field-specific reporting

Please select the one below that is the best fit for your research. If you are not sure, read the appropriate sections before making your selection.

☒ Life sciences ☐ Behavioural & social sciences ☐ Ecological, evolutionary & environmental sciences

For a reference copy of the document with all sections, see nature.com/documents/nr-reporting-summary-flat.pdf

Life sciences study design

All studies must disclose on these points even when the disclosure is negative.

Sample size	990
Data exclusions	No data exclusions
Replication	all attempts at replications were successful.
Randomization	All samples were allocated randomly.

Blinding	Investigators were binded to group allocation during data collection.

Reporting for specific materials, systems and methods

We require information from authors about some types of materials, experimental systems and methods used in many studies. Here, indicate whether each material, system or method listed is relevant to your study. If you are not sure if a list item applies to your research, read the appropriate section before selecting a response.

Materials & experimental systems

n/a	Involved in the study
☒	☐ Antibodies
☒	☐ Eukaryotic cell lines
☒	☐ Palaeontology and archaeology
☒	☐ Animals and other organisms
☒	☐ Human research participants
☒	☐ Clinical data
☒	☐ Dual use research of concern

Methods

n/a	Involved in the study
☒	☐ ChIP-seq
☒	☐ Flow cytometry
☒	☐ MRI-based neuroimaging
